# Comprehensive analysis of gene expression patterns of *hedgehog*-related genes

**DOI:** 10.1186/1471-2164-7-280

**Published:** 2006-10-31

**Authors:** Limin Hao, Robert Johnsen, Gilbert Lauter, David Baillie, Thomas R Bürglin

**Affiliations:** 1 Department of Biosciences and Nutrition, and Center for Genomics and Bioinformatics, Karolinska Institutet, SE-141 57 Huddinge, Sweden; 2 School of Life Sciences, Södertörns Högskola, Alfred Nobels Allé 7, SE-141 89 Huddinge, Sweden; 3 Department of Molecular Biology and Biochemistry, Simon Fraser University, Burnaby, B.C. Canada

## Abstract

**Background:**

The *Caenorhabditis elegans *genome encodes ten proteins that share sequence similarity with the Hedgehog signaling molecule through their C-terminal autoprocessing Hint/Hog domain. These proteins contain novel N-terminal domains, and *C. elegans *encodes dozens of additional proteins containing only these N-terminal domains. These gene families are called *warthog*, *groundhog*, *ground-like *and *quahog*, collectively called *hedgehog *(*hh*)-related genes. Previously, the expression pattern of seventeen genes was examined, which showed that they are primarily expressed in the ectoderm.

**Results:**

With the completion of the *C. elegans *genome sequence in November 2002, we reexamined and identified 61 *hh*-related ORFs. Further, we identified 49 *hh*-related ORFs in *C. briggsae*. ORF analysis revealed that 30% of the genes still had errors in their predictions and we improved these predictions here. We performed a comprehensive expression analysis using GFP fusions of the putative intergenic regulatory sequence with one or two transgenic lines for most genes. The *hh*-related genes are expressed in one or a few of the following tissues: hypodermis, seam cells, excretory duct and pore cells, vulval epithelial cells, rectal epithelial cells, pharyngeal muscle or marginal cells, arcade cells, support cells of sensory organs, and neuronal cells. Using time-lapse recordings, we discovered that some *hh*-related genes are expressed in a cyclical fashion in phase with molting during larval development. We also generated several translational GFP fusions, but they did not show any subcellular localization. In addition, we also studied the expression patterns of two genes with similarity to Drosophila *frizzled*, T23D8.1 and F27E11.3A, and the ortholog of the Drosophila gene *dally-like*, *gpn-1*, which is a heparan sulfate proteoglycan. The two *frizzled *homologs are expressed in a few neurons in the head, and *gpn-1 *is expressed in the pharynx. Finally, we compare the efficacy of our GFP expression effort with EST, OST and SAGE data.

**Conclusion:**

No bona-fide Hh signaling pathway is present in *C. elegans*. Given that the *hh*-related gene products have a predicted signal peptide for secretion, it is possible that they constitute components of the extracellular matrix (ECM). They might be associated with the cuticle or be present in soluble form in the body cavity. They might interact with the Patched or the Patched-related proteins in a manner similar to the interaction of Hedgehog with its receptor Patched.

## Background

The Hh signaling pathway is important for the development of animals from flies to vertebrates. It is an intercellular signaling pathway that regulates cell fate specification, cell proliferation and cell survival of different target cells [1]. In brief, the signaling cell produces a Hh precursor that is composed of the N-terminal domain (Hh-N), which is responsible for signaling, and the C-terminal Hint/Hog domain, where the proteolytic activity resides. Hh undergoes autocatalytic cleavage through its Hint domain and simultaneously anchors a cholesterol to the C terminus of Hh-N [2]. By further modification through addition of a palmitate at its N terminus, the mature signaling molecule Hh-N is generated. It is targeted to the cell membrane and tethered to it by lipid moieties. With the help of Dispatched the tethered Hh-N is released to the extracellular environment, where it can move several cell diameters to target cells. Molecules in the ECM, such as Dally-like, regulate the spread of Hh-N. In this way, Hh-N morphogens are distributed in a gradient that precisely patterns tissues. On the membrane of the target cell, the direct receptor Patched constitutively inhibits the activity of Smoothened (Smo). Upon binding to Hh-N, the inhibition of Smo is relieved and the signal transduces downstream through PKA and the Fu/SuFu/Costal-2 complex to the effector, Ci, a transcription factor [3].

In *C. elegans *there is no complete Hh signaling pathway, since it lacks a *bona fide *Hh, Smo and Fu/SuFu/Costal-2 complex. However, Dispatched, Patched and Ci are conserved [4]. One of the two Dispatched proteins, CHE-14, is involved in exocytosis [5]. The *patched *(ptc) and *patched*-related genes (*ptr*) have undergone substantial expansion in *C. elegans*; there are two *patched *genes, one *ptc *pseudogene, two *dispatched *(*che-14*, *ptd-2*), and 24 *patched*-related (*ptr*) genes [6]. *ptc-1 *is expressed in oocytes and involved in cytokinesis [6], and *ptr-7 *(*daf-6*) has been assigned a function in endocytosis during tubulogenesis [7]. *tra-1*, the ortholog of *Ci*, is involved in regulating sex determination [8]. Using RNA interference (RNAi), Zugasti et al. (2005) found that disruption of several *hh*-related genes as well as many *ptc *and *ptr *genes leads to molting defective phenotypes, suggesting that both *hh*-related genes and *ptc *and *ptr *genes might be involved in a common developmental pathway [9].

*C. elegans *has a hypodermis that ensheaths the entire body. During embryogenesis, the ectoderm is composed of six rows of cells that fuse with each other to form a few syncytia. In larvae, hyp1 to hyp6 envelope the head, the largest syncytium hyp7 constitutes the main body hypodermis, and hyp8 to hyp11 compose the tail. On the sides of the body there are two lateral hypodermal syncytia, the seam cells. All of these hypodermal cells secrete ECM components that form the cuticle, which forms the worm's exoskeleton. The cuticle, the hypodermis, and the underlying muscle and its basement membrane are anchored together by fibrous organelles to form a structure necessary for efficient locomotion of the worm. The openings of the worm, including the buccal cavity, sensory organs, excretory duct and pore, vulva and anus are formed by cells with epithelial properties that connect to the hypodermal syncytium. They are also covered by cuticle that connects to the body cuticle.

Hh is the only protein known to be modified by cholesterol through autocleavage mediated by its own Hint/Hog domain. However, although there is no *bona fide *Hh, the *C. elegans *genome encodes ten genes each with a conserved C-terminal Hint/Hog domain [10,11]. These proteins contain different types of N-terminal domains, and *C. elegans *encodes dozens of additional proteins containing only these N-terminal domains [4,11]. Based on the different N-terminal domains, these genes can be classified into four gene families – *warthog *(*wrt*), *groundhog *(*grd*), *ground-like *(*grl*), and *quahog *(*qua*). These four gene families are conserved in nematodes, but not in other phyla, and their gene products have predicted signal peptides for secretion [11]. Because a *hh *gene has recently been identified in cnidarians, the most parsimonious explanation for these nematode specific gene families is that they are evolutionarily derived from a *hh *gene in early nematode evolution [12], and that the loss of the Hog domain in many of the genes is a secondary event, as earlier postulated [11].

One of the Hint/Hog-containing peptides, WRT-1, has been shown to be autocleaved in vitro [13]. Previously, the gene expression patterns of eight *wrt *genes, eight *grd *genes and *qua-1 *were analyzed [11,14,15]. These genes were found to be mainly expressed in the hypodermis, seam cells, the excretory duct and pore, support cells of sensory organs, and some neuronal cells [11]. As a first step to gain insights into the function of the unstudied *hh*-related genes, we made a comprehensive analysis of the expression patterns of these genes using transcriptional GFP fusions.

## Results

### *C. elegans *contains 61 *hh*-related open reading frames (ORFs) and *C. briggsae *49

In November 2002, the *C. elegans *genome sequence was completely finished, with no gaps remaining. To determine the final complement of *hh*-related genes we performed comprehensive tblastn, blastp and PSI-blast searches of the *C. elegans *genome with all previously known *hh*-related genes [11]. Likewise, we searched the genome of *C. briggsae *for homologs. The previously unnamed *ground-like *genes were given the official designation *grl *and numbered. In our searches we identified four additional genes in *C. elegans *(*grl-24*, *grl-25*, *grl-26*, *grl-27*) (Table 1), yielding a total of 61 *hh*-related ORFs. 49 *hh*-related genes were retrieved from *C. briggsae *[see Additional file 1]. Three of the *C. elegans hh*-related ORFs are pseudogenes, two contain frame shifts (*grd-17p *and *grl-30p*) and one (*grl-32p*) is a duplication of only the last two exons of another grl gene. This was corroborated by consultation with the Genome Sequencing Consortium to verify that these aberrations are not sequencing errors.

**Table 1:** Definitive list of *hh*-related genes in *C. elegans *with prediction errors and expression summary.

Gene^†^	ORF	Hog	ORF prediction errors	Expression^‡^	EST clone No.	OST clone No.	SAGE hit	Links
*wrt-1*	ZK1290.12	+		ND, PY	1	1	+	[11]
*wrt-2*	F52E4.6			ND, PY	1	2	+	[11]
*wrt-3*	F38E11.7		Merged with upstream ORF	ND, PY	1	1	+	[11]
*wrt-4*	ZK678.5	+		ND, PY	10	1	+	[11]
*wrt-5*	W03D2.5			ND, PY	4	4	+	[11, 14]
*wrt-6*	ZK377.1	+		ND, PY	4	2	+	[11]
*wrt-7*	ZK1037.10	+	N- and C-term. wrong	ND, PN	0	2	+	[11]
*wrt-8*	C29F3.2	+		ND, PY	3	1	+	[11]
*wrt-9*	B0344.2		was 2 separate ORFs, with wrong exons	Y	0	0	+	Fig. 2A
*wrt-10*	ZK1290.8			Y	1	2	+	Fig. 2A, B, C, Table 4
*grd-1*	R08B4.1	+	N-term. wrong	N, PY	5	0	+	[11]
*grd-2*	F46B3.5	+	N-term. wrong	N, PN	3	1	+	[11]
*grd-3*	W05E7.1		merged with *grd-13*	ND, PY	15	0	+	[11]
*grd-4*	T01B10.1			N	0	3	+	
*grd-5*	F41E6.2			Y, PY	10	0	+	Fig. 2B, C, Table 2, 4, [11]
*grd-6*	T18H9.1			Y, PY	6	1	+	PVT neuron, un-identified cells, [11]
*grd-7*	F46H5.6			Y, PY	0	1	+	Fig. 2B, [11]
*grd-8*	C37C3.4			Y, PN	0	2	-	Table 5, [11]
*grd-9*	C04E6.6		N- and C-term. wrong	N, PN	0	0	+	
*grd-10*	F09D12.1			Y	9	2	+	Fig. 1B
*grd-11*	K02E2.2	+		N	0	1	+	
*grd-12*	F02D8.2			Y	3	2	+	Fig. 2C, Table 2
*grd-13*	W05E7.3		merged with *grd-3*	Y	4	0	+	Fig. 2B, C, Table 3
*grd-14*	T01B10.2		N-term. wrong	Y	10	3	+	Fig. 2A, B, C
*grd-15*	Y87G2A.15		N-term. wrong	Y	0	0	+	Un-identified cells in the head and tail
*grd-16*	Y69A2AL.1		ORF totally wrong, basically not present	Y	0	4	-	Fig. 1B, Table 5
*grd-17*p	Y102A5c.34			ND	0	0	+	
*hog-1*	W06B11.4	+		Y	1	3	+	Fig. 2C a few un-identified cells in the head
*qua-1*	T05C12.10	+		PY	11	2	+	[15]
*grl-1*	C24G6.7			Y	0	2	+	Table 5
*grl-2*	T16G1.8			Y	1	1	+	Table 5
*grl-3*	K03B8.7			N	0	0	+	
*grl-4*	F42C5.7		N-term., 1. exon wrong	Y	5	2	+	Fig. 2A, B, C, Table 2, 4
*grl-5*	Y47D7A.5			Y	15	2	+	Fig. 2A, C
*grl-6*	K10C2.5			Y	0	3	+	Table 5
*grl-7*	T02E9.2			Y	24	1	+	Fig. 2B
*grl-8*	ZC487.5			Y	0	1	+	Fig. 2C, Table 5
*grl-9*	ZC487.4			Y	2	2	+	Table 3
*grl-10*	C26F1.5			Y	10	1	+	Fig. 2B, C, Table 2
*grl-11*	ZK512.9			Y	0	2	+	Table 3
*grl-12*	F28A12.2		N-term. too long	Y	1	0	+	Table 5
*grl-13*	F32D1.4		N-term. wrong prediction	Y	0	0	+	Table 5
*grl-14*	T03D8.4			Y	1	2	+	Table 4
*grl-15*	Y75B8A.20			Y	5	0	+	Fig. 2A, B, Table 2
*grl-16*	Y65B4BR.6			N	22	3	+	
*grl-17*	C56A3.1			Y	0	1	+	Fig. 2A, C, Table 3, 5
*grl-18*	T05C3.4		N-term. wrong	Y	0	1	+	Table 5
*grl-19*	R02D3.6			Y	1	0	+	Excretory duct cell
*grl-20*	C23H5.9			N	22	2	+	
*grl-21*	ZC168.5			Y	4	4	+	Fig. 2A
*grl-22*	W03A5.3			ND	0	1	+	
*grl-23*	E02A10.2			N	0	2	+	
*grl-24*	F11E6.2			N	2	0	+	
*grl-25*	ZK643.8		N-term. wrong	Y	2	0	+	Table 2
*grl-26*	K02D7.6		C-term. exon missed	Y	1	0	-	Table 4
*grl-27*	F40C5.3			Y	1	2	+	Fig. 2B, Table 4
*grl-28*	T24A6.15			Y	0	2	+	Table 5
*grl-29*	T24A6.18			Y	0	0	-	Fig. 2C, Table 5
*grl-30*p	T24A6.3			ND	0	3	-	
*grl-31*	T24A6.19			Y	0	3	-	Table 2
*grl-32*p	T24A6.21		wrongly merged with other ORF	ND	0	0	-	

61 total (3 pseudo)			18 (30%) wrong prediction	47	36	43	54	<-expression positive totals

The ORF prediction errors column briefly describes errors found in the gene prediction at the onset of the project; the expression column indicates whether expression was seen. The p after a gene name indicates pseudogenes: *grd-17 *(N-term. frameshift), *grl-30 *(N-term. frameshift), *grl-32 *(duplication of last two exons of *grl-27*). The Hog column indicates which genes have a Hint/Hog domain. Expression column key: Y, expression; N, no expression; ND, not determined, no transgenic line/PCR construct; PY, expression in previous work [11]; PN, no expression in previous work [11]. The EST column indicates how many EST clones were found for that gene, the OST column how many OSTs were obtained. The SAGE hit column summarizes SAGE data: a plus indicates at least one hit. The Links column cites previous relevant publications and provides links to figures and tables in this paper.

**Figure 1:**
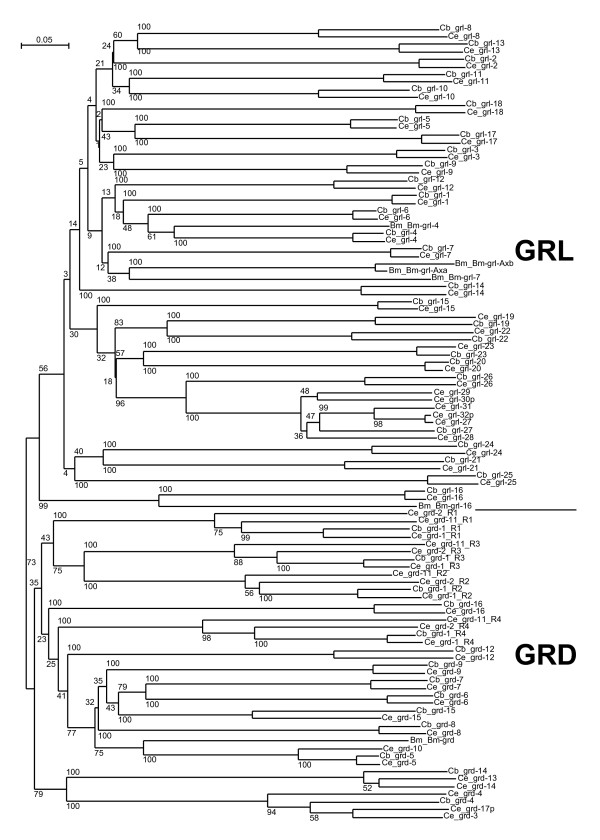
**Phylogenetic tree of *C. elegans *and *C. briggsae *Ground and Ground-like domains**. Multiple sequence alignment of Ground and Ground-like domains were generated using MAFFT [see Additional file 3]. For *grd-1*, *grd-2 *and *grd-11 *the four Ground domains were extracted manually prior to alignment; the R1 to R4 postscripts indicate which repeat it is. Neighbor joining was performed on the aligned sequences using the default parameters of Clustal_X. Results of 100 bootstrap trials are shown. In addition to the *C. elegans and C. briggsae *sequences, some *Brugia malayi *(Bm) sequences from previous work were also included [4].

**Figure 2:**
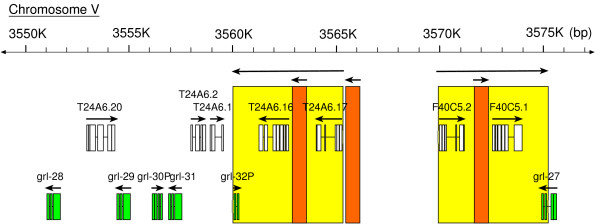
***C. elegans grl *gene cluster on chromosome V**. The ORFs in the approximately 25 kb large genomic region are indicated. The *grl *ORFs are highlighted in green. The yellow box shows an inverted repeat of about 5 kb, and the orange box indicates a small repeat of about 1 kb. Note that the duplication that created the large repeat gave rise to *grl-32p*.

In the course of this analysis we also checked the ORF predictions. We compared orthologous genes between *C. elegans *and *C. briggsae *as well as similarity between closely homologous *C. elegans *genes (see Methods). Furthermore, we verified N-terminal sequences using the SignalP server to determine, whether a signal sequence for protein export was present. Despite the fact that a previous publication [11] was available defining the features of these genes, we found that 30% of the genes in Wormbase in 2003 still had errors in their ORF predictions. In many instances this was in the N-terminal exons (Table 1). After correcting the ORFs, we found that all genes except a gene that we named *hog-1 *encoded a good signal sequence for protein export according to SignalP. *hog-1 *encodes only the C-terminal autocatalytic domain and no trace of an upstream N-terminal domain could be identified, even by comparison with the orthologous genomic region of *C. briggsae*. The evolutionary conservation of *hog-1 *suggests that this gene is not simply a pseudogene that lost the N-terminal region. Our improved predictions were submitted to Wormbase and the new nomenclature can be found in WS release 153 and later releases. Since we made these predictions, additional EST data has become available – primarily from a recent batch of ESTs from the Exelixis *Caenorhabditis elegans *EST project – and ten of our ORF predictions have now been independently verified. *C. elegans *has 12 *hh*-related ORFs more than *C. briggsae*. This is entirely due to *C. elegans *specific gene duplications. *C. briggsae *has one *wrt *gene less. *C. elegans wrt-7 *and *wrt-8 *are very similar and lie next to each other on the chromosome, indicating a tandem duplication event, and only a single co-ortholog exists in *C. briggsae*. For the *grd *and *grl *genes we performed phylogenetic analysis to verify orthology (Fig. 1). Three *grd *genes, *grd-1*, *grd-2*, and *grd-11 *each encode four Ground domains, and there is only a single corresponding co-ortholog in *C. briggsae*. Of the three *C. elegans *genes, *grd-1 *is the most conserved, while *grd-2 *and *grd-11 *are more divergent. A small cluster of three *grd *genes (*grd-13*, *grd-3*, *grd-10*, and *grd-14*, *grd-4*, *grd-5*) is duplicated in *C. elegans *[11], and only one corresponding cluster is present in *C. briggsae*. *C. elegans grd-5 *and *C. briggsae grd-5 *have separated from their respective clusters, however. The *C. elegans *pseudogene *grd-17p *is a recent duplicate of *grd-3*. In the case of the *grl *genes, most have one to one corresponding orthologs between *C. elegans *and *C. briggsae*. However, in *C. elegans *we find one *grl *gene cluster that has expanded from a single gene into six ORFs, two of which are pseudogenes (Fig. 2). This locus has other hallmarks of recent gene duplication activity, such as a 5 kb large inverted repeat, which duplicated the last two exons of *grl-27 *to give rise to *grl-32p *(Fig. 2). Contained within the big repeat is a smaller duplicated segment, which could have transposed regulatory elements from F40C5.1 to T24A6.17 (both not *hh*-related genes).

### *hh*-related genes are expressed in the hypodermis or epithelial derived cells

Previously, the expression patterns of eight *wrt *genes (*wrt-1 *to *wrt-8*) and eight *grd *genes (*grd-1 *to *grd-3 *and *grd-5 *to *grd-9*) [11,14], and the single copy gene *qua-1 *have been investigated [15]. Here, we used GFP fusions to examine the expression patterns of the two remaining genes in the *wrt *family, all 16 genes in the *grd *family, and 30 genes in the *grl *gene family, and *hog-1*. Our newly generated ORF predictions served to determine the proper start codon. Most promoter::GFP fusions were generated using PCR stitching with the putative intergenic regulatory region, and generating one or two transgenic lines for each construct (see Methods). Such constructs might miss some regulatory elements, for example those in introns, and other limitations of the methodology have been pointed out by McKay et al. [16]. However, in our case a distinct advantage of the promoter-GFP constructs was that the signal peptides for secretion were avoided and therefore the expressing cells could be identified more easily. Three predicted genes proved to be pseudogenes (*grd-17p*, *grl-30p *and *grl-32p*) and were omitted in the present study (Table 1). For two genes (*grd-3 *and *grl-22*) we failed to obtain GFP reporter constructs. All in all, we obtained GFP expression constructs for 47 *hh*-related genes that were analyzed in 79 corresponding transgenic worm strains [see Additional file 2]. Observation of these transgenic worms revealed that out of 47 genes, ten did not show any detectable expression by the approach we used. These genes were *grd-1*, *grd-2*, *grd-4*, *grd-9*, *grd-11*, *grl-3*, *grl-16*, *grl-20*, *grl-23 *and *grl-24 *(Table 1). *grd-1 *has been found to be expressed previously [11], and all ten genes have corresponding EST and/or OST (open-reading-frame sequence tag) clones, and/or have been detected in SAGE analysis (Table 1), so it is likely that they are expressed in vivo, but not detected by our approach.

**Figure 3:**
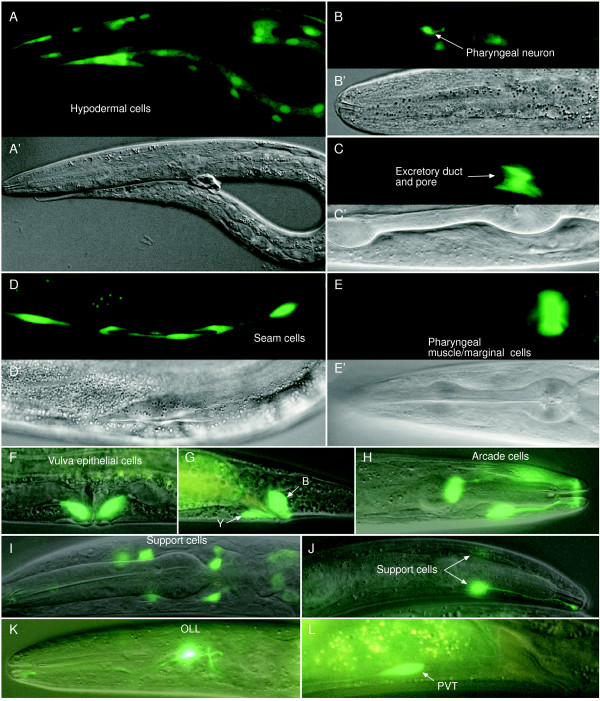
**Representative expression patterns of *hh*-related genes**. (A, A') P*wrt-9*::GFP is expressed in all hypodermal cells, except seam cells. (B, B') P*grd-5*::GFP is expressed in a pharyngeal neuronal cell. (C, C') P*grl-2*::GFP is expressed in the excretory duct and pore cells. (D, D') P*grd-10*::GFP is expressed in seam cells; the mid-body region is shown. (E, E') P*grl-9*::GFP is expressed in muscle or marginal cells in the terminal bulb of the pharynx. (F) P*grd-12*::GFP is expressed in two vulval epithelial cells. (G) P*grd-12*::GFP is expressed in two epithelial cells. (H) P*grl-27*::GFP is expressed in arcade cells. (I) P*grd-8*::GFP is expressed in neuronal support cells. (J) P*grl-18*::GFP is expressed in two support cells. The process of one cell is shown. (K) P*grl-8*::GFP is expressed in two OLL neurons. (L) P*grl-6*::GFP is expressed in the PVT neuron. A', B', C', D' and E' are differential interference contrast (DIC) images corresponding to the fluorescent images in A, B, C, D and E, respectively. Images in F-L are overlapping of fluorescent and DIC images.

Here, we summarize the expression patterns of the 37 *hh*-related genes that showed expression. We find that these genes are expressed in one or a few of the following tissues: the hypodermis (Fig. 3A, A'), seam cells (Fig. 3D, D'), vulval epithelial cells (Fig. 3F), rectal epithelial cells (Fig. 3G), arcade cells (Fig. 3H), excretory duct and pore cells (Fig. 3C, C'), support cells of the sensilla (Fig. 3I and 3J), pharyngeal muscle or marginal cells (Fig. 3E, E'), and different types of neurons (Fig. 3B, B', K and 3L).

### The temporal expression of *hh*-related genes in the hypodermis, seam cells and rectal epithelial cells

**Figure 4:**
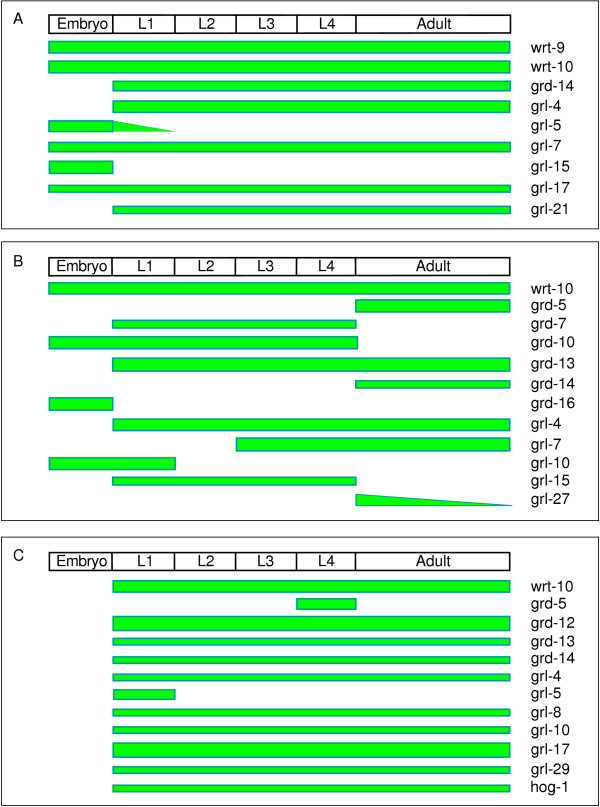
**Schematic of the temporal and spatial expression patterns of *hh*-related genes**. (A-C) Summarized spatial and temporal expression levels of *hh*-related genes in hypodermal cells (A), seam cells (B) and rectal epithelial cells (C). The width of the green bar represents the approximate GFP signal intensity.

Out of the 37 *hh*-related genes showing expression, nine genes (*wrt-9*, *wrt-10*, *grd-14*, *grl-4*, *grl-5*, *grl-7*, *grl-15*, *grl-17 *and *grl-21*) are expressed in the hypodermis (Fig. 3A, A' and 4A), 12 genes (*wrt-10*, *grd-5*, *grd-7*, *grd-10*, *grd-13*, *grd-14*, *grd-16*, *grl-4*, *grl-7*, *grl-10*, *grl-15 *and *grl-27*) in the seam cells (Fig. 3D, D' and 4B), and 12 genes (*wrt-10*, *grd-5*, *grd-12*, *grd-13*, *grd-14*, *grl-4*, *grl-5*, *grl-8*, *grl-10*, *grl-17*, *grl-29 *and *hog-1*) in the rectal epithelial cells (Fig. 3G and 4C). By observing worms at different developmental stages we determined the temporal expression and the relative intensity of the GFP signal. In the hypodermis, there are two *hh*-related genes (*grl-5 *and *grl-15*), which are mainly expressed in the embryo, three genes (*grd-14*, *grl-4 *and *grl-21*) only expressed in larval and adult stages, and four genes (*wrt-9*, *wrt-10*, *grl-7 *and *grl-17*) expressed from embryo stage through adulthood (Fig. 4A). The temporal expression of *hh*-related genes in seam cells is more variable (Fig. 4B). Four genes (*wrt-10*, *grd-10, grd-16 *and *grl-10*) start expression in the embryonic stage, but end at different postembryonic stages. Seven genes (*wrt-10*, *grd-5*, *grd-13*, *grd-14*, *grl-4*, *grl-7 *and *grl-27*) start expression at different stages, but all maintain expression throughout adulthood. One gene (*grl-15*) is only expressed in larval stages, and expression of another gene (*grl-27*) was found to decrease in old adults. In rectal epithelial cells, *grl-5 *is expressed only in the first larval stage (L1) and *grd-5 *only in the fourth larval stage (L4) (Fig. 4C). All other genes start their expression at L1 and maintain it through adulthood. The expression levels of three genes (*wrt-10*, *grd-12 *and *grl-17*) are higher than other genes in the rectal epithelial cells (Fig. 4C).

### The spatial expression of *hh*-related genes in vulval epithelial cells, pharyngeal muscle cells, arcade cells and neuronal support cells

The expression of *hh*-related genes shows spatial differences, i.e. they are expressed in different organs and/or tissues. Within a single organ, e.g., the vulva, we observed expression in different vulval cell types. Seven *hh*-related genes are expressed in the vulval epithelial cells (a representative image is shown in Fig. 3F). There are three genes (*grl-4*, *grl-10 *and *grl-25*) expressed in VulA, four (*grd-5*, *grl-4*, *grl-10 *and *grl-15*) in VulB, two (*grd-12 *and *grl-15*) in VulC, two (*grd-5 *and *grl-15*) in VulD, one (*grl-15*) in VulE, and one (*grl-31*) in vulF (Table 2).

**Table 2:** *hh*-related genes expressed in vulval epithelial cells.

Gene	Worm strain	VulA	VulB	VulC	VulD	VulE	VulF
*grd-5*	BC12828, BC12591		+		+		
*grd-12*	BC12962			+			
*grl-4*	BC12855	+	+				
*grl-10*	BC12880, BC13003	+	+				
*grl-15*	BC12984		+	+	+	+	
*grl-25*	BC15248	+					
*grl-31*	BC20024						+

Four genes are expressed in the pharyngeal muscle or marginal cells (a representative image is shown in Fig. 3E, E'). One gene (*grd-13*) is expressed in the pro- and metacorpus and isthmus, and three genes (*grl-9*, *grl-11 *and *grl-17*) in the terminal bulb of the pharynx (Table 3). Six genes are expressed in the arcade cells (a representative image is shown in Fig. 3H). Three of these (*wrt-10*, *grl-26 *and *grl-27*) are expressed in both anterior and posterior arcade cells, and three (*grd-5*, *grl-4 *and *grl-14*) only in anterior arcade cells (Table 4).

**Table 3:** *hh*-related genes expressed in pharyngeal cells.

Gene	Worm strain	Pharyngeal cells
*grd-13*	BC15195	Pro- and meta-corpus, isthmus
*grl-9*	BC15288	Terminal bulb, weak in larvae and strong in adults
*grl-11*	BC12881	Terminal bulb (pm6).
*grl-17*	BC15275, BC15276	Terminal bulb

**Table 4:** *hh*-related genes expressed in arcade cells.

Gene	Worm strain	Arcade cells
*wrt-10*	TB1616, TB1618	Anterior and Posterior arcades
*grd-5*	BC12828, BC12951	Anterior arcade cells
*grl-4*	BC15274	Anterior arcade cells
*grl-14*	BC15224	Anterior arcade cells
*grl-26*	BC20127	Anterior and Posterior arcades
*grl-27*	BC15279, BC15280	Anterior and Posterior arcades

Twelve genes (*grd-8*, *grd-16*, *grl-1*, *grl-2*, *grl-6*, *grl-8*, *grl-12*, *grl-13*, *grl-17*, *grl-18*, *grl-28 *and *grl-29*) are expressed in cells whose bodies are located around the anterior bulb of the pharynx and which have processes extending anteriorly (Fig. 3I and 3J). Most of the socket and sheath cell bodies of the amphids, IL, OL, and the cephalic sensilla are located around the metacorpus. We did not precisely identify which support cells express each gene, but judged by the position of the cell bodies and their processes, we expect them to be support cells – in most cases socket cells of IL, OL, cephalic and/or amphid sensilla (Table 5).

**Table 5:** *hh*-related genes expressed in neuronal support cells.

Gene	Worm strain	Neuronal support cells
*grd-8*	BC12538	Socket cells of IL or OLQ
*grd-16*	BC15226	Socket cells of IL or OLQ
*grl-1*	BC15287	Socket cells of IL or OLQ
*grl-2*	BC12852	Socket cells of amphids, IL or OLQ
*grl-6*	BC12856	Socket cells of IL or OLQ
*grl-8*	BC15274	Socket cells of IL or OLQ
*grl-12*	BC20023	Socket cells of amphids
*grl-13*	BC15244	Socket cells of IL or OLQ
*grl-17*	BC15276	Socket cells of IL or OLQ
*grl-18*	BC15230	Socket cells of IL or OLQ
*grl-28*	BC15281	Sheath cell of amphids
*grl-29*	BC15282, BC15283	Socket cells of IL or OLQ

### Expression of *hh*-related genes in other cell types

Four genes (*wrt-10*, *grd-2*, *grd-6 *and *grl-19*) are expressed in the excretory duct and pore cells (Fig. 3C, C'). Some *hh*-related genes have been found to be expressed in different types of neurons. *grd-8 *is strongly expressed in OLLL and OLLR neurons (Fig. 3K). *grd-5 *is expressed in a pharyngeal neuron in the metacorpus extending a neurite anteriorly and an axon posteriorly (Fig. 3B, B'). *grl-6 *is expressed in the PVT cell (Fig. 3L). Some genes (e.g., *grl-1*) are expressed in neurons of the lumbar ganglion, but the specific neurons were not identified (data not shown). We also found that some genes (e.g., *grd-16 *and *grl-19*) showed expression in the intestine, usually stronger in the cells at the terminal regions (data not shown).

### Cyclical changes of expression levels of some *hh*-related genes

**Figure 5:**
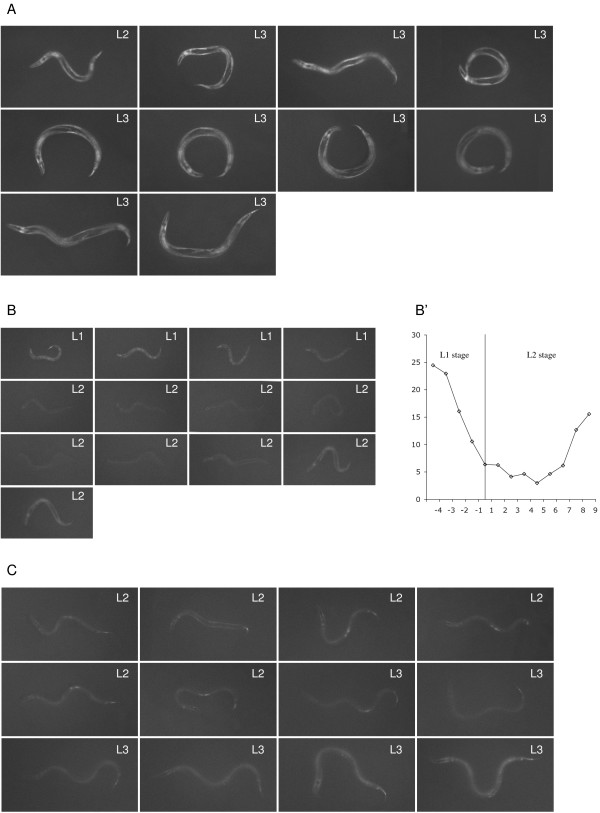
**Time-lapse recordings of the expression level of *hh*-related genes**. (A) One transgenic larva carrying P*grl-4*::GFP was recorded starting from late L2 to late L3. There is no apparent change in the expression level before and after molt. (B, B') One transgenic larva carrying P*wrt-9*::GFP was recorded starting from middle L1 to late L2. The GFP signal is high 3–4 hr before the L1 molt and then goes down and reaches a valley 3–4 hr after the L1 molt and then increases again. A graph representing the intensity change was generated from the raw image data as described in Methods. (C) One transgenic larva carrying P*wrt-10*::GFP is recorded starting from mid-L2 to mid-L3. The GFP signal is high 5–6 hr before the L2 molt. It decreases and reaches its lowest level around 2 hr after the L2 molt and then increases again. In all the three panels, the time interval between images is 1 hr.

Because of the large syncytium of the hypodermis and strong GFP signals, it was possible to observe the gene expression levels of several genes dynamically using time-lapse recording of single live transgenic GFP expressing worms under a dissecting fluorescent microscope. Seven out of nine *hh*-related genes expressed in the hypodermis, show expression in larvae and adult stages. But only five of these have strong enough GFP signals that allowed time-lapse recordings. The expression level of the P*grl-4*::GFP transgene is constant from larvae to adults (Fig. 5A). However, four *hh*-related genes display cyclical changes in expression levels during larval development. Using *wrt-9 *as an example, we observed that the expression level of P*wrt-9*::GFP reached its peak around four hours before each molt, lowered during molting and reached the lowest level around five hours after molting. This cycle repeated again in the next larval stage (Fig. 5B, B' and data not shown). The expression of two other transgenes (P*grd-14*::GFP and P*grl-7*::GFP) showed similar patterns (data not shown). The expression level of *wrt-10 *also displays cyclical changes, but the timing is shifted by about two hours, i.e. the time for maximal and minimal expression is two hours earlier than that of the other three genes (Fig. 5C).

### Localization of WRT-10, WRT-9 and GRL-4 revealed by translational GFP fusions

**Figure 6:**
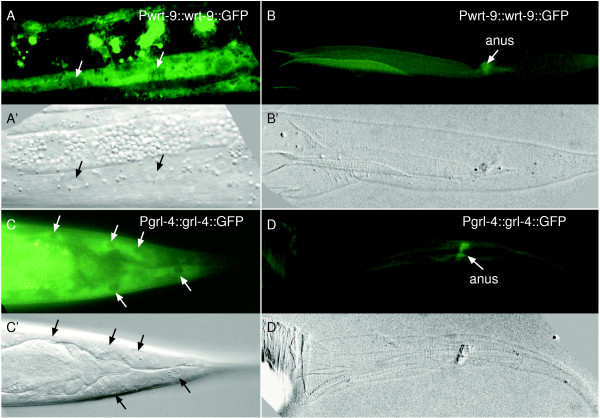
**Location of two proteins WRT-9 and GRL-4 revealed by translational GFP fusions**. (A, A') Expression of the P*wrt-9*::*wrt-9*::GFP in the hypodermis in the body region (arrows point to the nuclei of the hypodermal cell). (B, B') The GFP-tagged WRT-9 protein is not associated with the shed old cuticle (seen as a faint autofluorescent outline) during molting (the arrow points to the anus). (C, C') P*grl-4*::*grl-4*::GFP is weakly expressed in the hypodermis. Tail region is shown (arrows point to the nuclei of the hypodermal cells). (D, D') The GFP-tagged GRL-4 protein is not associated with the shed cuticle (the arrow points to the anus). A'-D' are DIC images corresponding to the fluorescent images A-D, respectively.

The oscillatory expression levels of some *hh*-related genes prompted us to test whether some of these proteins are associated with the cuticle like QUA-1; we have previously shown that QUA-1 is associated with the cuticle and that QUA-1::GFP can be seen in the shed cuticle during molting [15]. We made translational GFP fusions to the full-length coding sequences of *wrt-10 *and *grl-4*, and only the signal peptide region of *wrt-9*, driven by their own promoter. The transgenic worms carrying P*wrt-10*::*wrt-10*::GFP did not show any GFP signal. The two transgenic lines for *wrt-9 *and *grl-4 *exhibited GFP signal only in the hypodermis (Fig. 6A, A' and 6C, C'). We observed the molting process of live transgenic *wrt-9 *and *grl-4 *lines, but were not able to detect WRT-9::GFP or GRL-4::GFP protein outside the hypodermis or associated with the detached cuticle (Fig. 6B, B' and 6D, D'). Further, neither of these GFP fusions seemed to be associated with any obvious structure in the ECM. These fusion proteins might be secreted to the body cavity like WRT-5 [14], where we may have missed weak, diffuse GFP signals, or they might be very soluble and not stay attached to the ECM.

### Expression of orthologs of other components of the Hh signaling pathway

**Figure 7:**
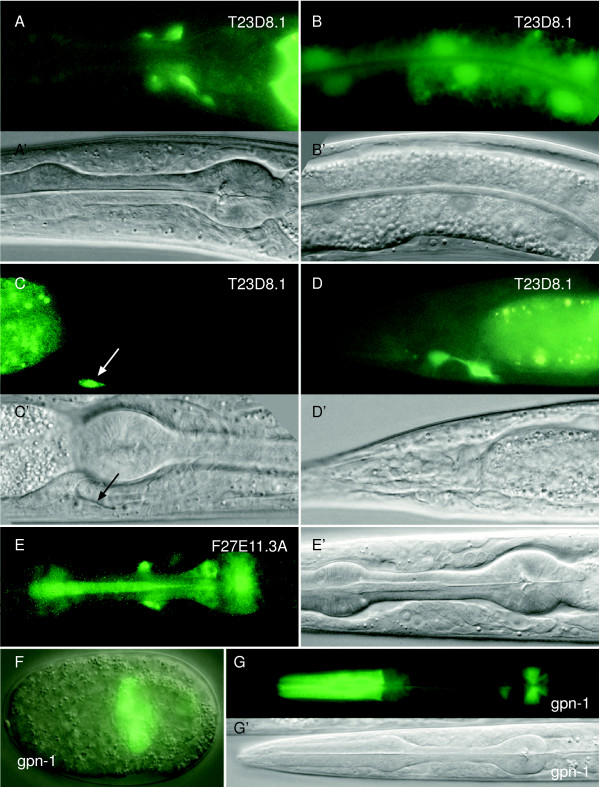
**Expression patterns of two *frizzled *genes T23D8.1 (*mom-5*) and F27E11.3A (*cfz-2*), and *gpn-1***. (A, A'-D, D') Expression of the *frizzled *homolog T23D8.1 monitored using GFP. P_T23D8.1_::GFP is expressed in a few cells of the nerve ring (A, A'). Further, it is expressed in the intestine (B, B'), one cell on the ventral side of the terminal bulb of the pharynx (arrow in C, C') and two cells around the anus (D, D'). (E, E') P_F27E11.3A_::GFP is expressed in three cells around the nerve ring, two cells in the terminal bulb of the pharynx, and also weakly in the pharynx. (F) P*gpn-1*::GFP is expressed in the pharyngeal primordium in embryos (F, merger of the DIC and fluorescent images). (G, G') P*gpn-1*::GFP is also expressed in the pharynx in larvae and adults, L1 stage shown. A'-E' and G' are DIC images corresponding to the fluorescent images in A-E and G, respectively.

*smo*, a member of the *frizzled *gene family, is an indispensable component for Hh signal transduction. The *C. elegans *genome encodes four genes with homology to *frizzled*. Alignment of these *frizzled *homologous genes with the *frizzled *gene sequences from other phyla showed that none of the *C. elegans frizzled *genes can be identified as being orthologous to *smo *(data not shown). Since two out of four *C. elegans frizzled *genes had already been characterized [17,18], we determined the expression pattern of the two remaining *frizzled *homologs, T23D8.1 and F27E11.3A, which now have been named *mom-5 *and *cfz-2*, respectively [19-21]. As shown in Fig. 7, T23D8.1 is expressed in a few cells in the nerve ring, putatively identified as neurons (Fig. 7A, A'). In addition, there is also expression in the intestine (Fig. 7B, B'), in one cell on the ventral side of the terminal pharyngeal bulb (Fig. 7C, C') and two cells around the anus (Fig. 7D, D'). F27E11.3A is expressed in three cells around the nerve ring, possibly neurons, in two cells in the posterior bulb of the pharynx, and in the pharyngeal marginal or muscle cells (Fig. 7E, E'). This observation is consistent with the report about *cfz-2 *[21]. However, we failed to see the expression in embryos, probably because of the low expression levels.

Hh requires heparan-sulfate modified proteoglycans for efficient movement through the ECM from the producing cells to the target cells. In *C. elegans*, *gpn-1 *encodes a homolog of Drosophila Dally-like, a heparan sulfate proteoglycan that is orthologous to mammalian glypicans [4]. We observed the expression pattern of *gpn-1 *and found that it is exclusively expressed in the pharyngeal muscle or marginal cells from the embryonic comma stage to adults (Fig. 7F and 7G, G').

## Discussion

### *hh*-related gene expression data

In this study we have updated the list of *hh*-related genes in *C. elegans *and also identified the corresponding orthologs in *C. briggsae*. This revealed that there has been gene amplification as well as pseudogene generation in *C. elegans*. Analysis of the ORFs revealed that 30% of the *hh*-related genes in *C. elegans *had prediction errors, mainly in the N-terminus, although in *C. elegans *relatively good gene predictions can be achieved, because of the small genome size, mostly small introns, and well-conserved splice sites. We noticed that in 10 ORFs with errors, no corresponding OSTs are available, and the errors in prediction may be directly responsible for the failure to obtain OSTs. This highlights the need for better knowledge of domain and gene structure, which can be combined with experimental methods, to refine and improve ORF predictions. This is of particular importance for other organisms that have much larger genes and introns, such as mammals. Many of our predictions have now been validated by new ESTs, indicating that careful bioinformatics analysis which takes into account multiple independent features (cross-species conservation, signal peptide, sequence motifs, consistent gene structure within a gene family), can indeed lead to improved predictions.

Our high-throughput reporter::GFP analysis is subject to the standard caveats that apply to transcriptional GFP reporter constructs in general [16]. In particular, the intergenic fragment containing the putative promoter may not contain all regulatory elements; for example, some may reside in introns. Further, GFP reporters are usually not expressed in the germ line. Generating primarily methionine fusions as we did has an important advantage: because there is no signal peptide for secretion, the GFP will remain in the cell where it is produced, and cells can be identified. Secreted GFP may be difficult to visualize and may diffuse to other cells, for example the coelomocytes, as we observed in the case of *wrt-5 *[14].

**Figure 8:**
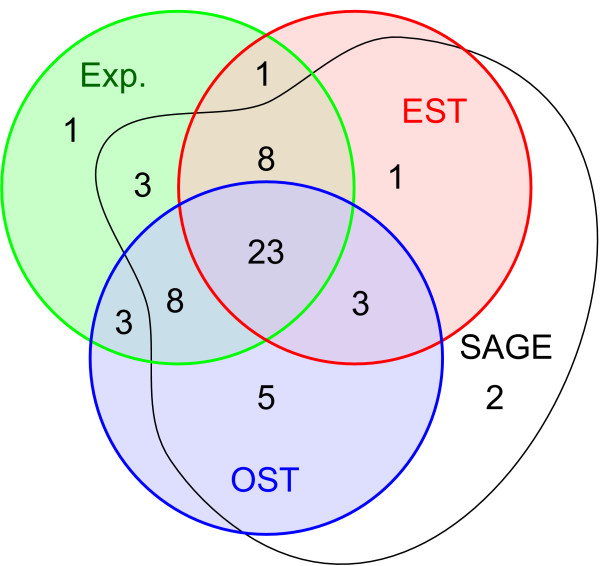
**Venn diagram summarizing gene expression data**. Number of genes detected with different experimental methods, summarized from Table 1. Information for pseudogenes is not included. GFP/lacZ expression (Exp.) data are shown in the green circle, EST data in the red circle, OST data in the blue circle, and SAGE data in the black outline.

Keeping in mind these caveats, this study, together with our previous work [11,14,15], provides a comprehensive analysis of the expression patterns of the *hh*-related genes. Out of the 61 ORFs, three pseudogenes and *grd-3 *and *grl-22 *were not included. For a further ten, no expression was observed. We have compiled EST, OST and SAGE data for all the *hh*-related ORFs to obtain independent verification about gene expression, in particular for those cases where we saw no GFP (Table 1), and the results are summarized in a Venn diagram (Fig. 8).

Of the 58 presumed functional ORFs, 42 ORFs are supported by at least three independent methods. 21 (36%) genes have no corresponding EST, and for 11 genes no lacZ or GFP expression was observed in this and previous studies. However, for four genes that have neither EST nor OST support we obtained GFP expression data, and one of these, *grl-29*, has no SAGE support either.

Four genes, for which we failed to observe GFP expression, are represented in the EST set, indicating that the transcripts are not rare. Particularly noteworthy are *grl-16 *and *grl-20*, since they both have many EST clones. The first exon of *grl-20 *is small and the first intron is over 1 kb in length. Therefore, essential regulatory elements could reside in this intron. A few reporter constructs, such as for *grd-1 *and *grd-4*, may fail to express, because they may be in an operon with upstream genes, and the intergenic fragments would not suffice. Reporter constructs and ESTs are perhaps the most reliable indicator of gene expression. The combined data show that only for seven genes out of 58 neither GFP/lacZ nor EST data is available. Five genes (*wrt-7*, *grd-4*, *grd-11*, *grl-22*, *grl-23*) are found both in OSTs and SAGE, suggesting that they are expressed, but possibly at low levels. *grd-9 *and *grl-3 *are only supported by SAGE data, *grd-9 *with multiple hits in multiple samples, but *grl-3 *with only a few hits. Two of the pseudogenes, *grd-17p *and *grl-30p*, are indicated to be transcribed, one by SAGE, and one through OSTs. Since these are recently duplicated genes, some promoter activity may still be remaining. In fact all duplicated *grl *genes in the cluster (*grl-27 *to *grl-31*) are expressed, but their expression patterns have diverged. Our comprehensive analysis of all the expression patterns shows that the vast majority of these genes are expressed in the hypodermis or in cells with epithelial properties. Noticeably, these cells are located on the outer surface of the worm body or line the openings of the animal. Many of these cells are involved in secreting cuticle components that line their surface, which is in direct contact with the environment. Understanding the function of these genes may not be trivial, since the overlapping of expression patterns suggests that there may be functional redundancy.

### The products of the *hh*-related genes might be components of or function in the ECM

During *C. elegans *embryonic development, the embryo elongates from an oval cell bundle to a four-fold vermiform shape. To maintain the shape the hypodermis secretes the cuticle that forms the worm's exoskeleton. During post-embryonic development, *C. elegans *renews its cuticle four times – one at each molt. The cuticle is composed of major structural components, i.e. cuticlins and collagens. All the cuticular components are secreted by the underlying hypodermal cells. Along the two lateral sides of the worm there are two special protruding structures, called alae, some components of which are secreted by the underlying seam cells. Fourteen *hh*-related genes are expressed in the hypodermis and 14 genes in the seam cells ([11] and Fig. 3 and 4). Since all of the *hh*-related genes have a predicted signal peptide for secretion, except *hog-1*, it is likely that their gene products are secreted. In addition, all of these proteins have multiple conserved cysteine residues that can often be found in secreted molecules and are expected to form disulfide bonds. Indeed, we have discovered that one of the *hh*-related genes, *qua-1*, is expressed in the hypodermis, and QUA-1 is secreted and associated with the cuticle [15]. Another example is WRT-5, which is expressed in seam cells during embryogenesis and is also secreted. However, in this case the protein was not localized to the cell surface, but diffused inside the embryonic egg shell and in larvae it was secreted into the pharyngeal lumen [14]. Despite the lack of localization of WRT-5 protein to the ECM, *wrt-5 *mutants display aberrant cell junctions, indicating that WRT-5 functions in the ECM [14].

The cycling expression pattern of some *hh*-related genes implies that their proteins play roles during larval development, particularly molting. Not only the main body, but also all the major openings of *C. elegans*, including the buccal cavity, pharyngeal lumen, vulva, rectum, excretory duct and pore, and openings of sensory organs are lined by cuticles. These cuticles are secreted by the underlying cells that are generally of epithelial nature. Anterior to the buccal cavity, the lip cuticle is secreted by three specialized rings of hypodermal cells hyp1–3. The buccal cuticle is secreted by the pharyngeal epithelium, and the arcade cells secrete the bridging cuticle that connects the lip and the buccal cuticles. The cuticle lining the pharynx is secreted by the pharyngeal muscle or marginal cells. Our discovery that there are six *hh*-related genes expressed in arcade cells (Fig. 3H and Table 4), four in pharyngeal muscle and marginal cells (Fig. 3E and Table 3) suggests that these gene products might also be secreted to the pharynx cuticle or lumen as is WRT-5 [14].

The cuticles at the openings of the vulva, rectum and excretory duct and pore are secreted by the underlying vulval epithelial cells, rectal epithelial cells and excretory duct and pore cells. Our results show that there are eight *hh*-related genes expressed in vulval epithelial cells, 11 in rectal epithelial cells and three in the excretory duct and pore cells. The products of these genes are also secreted and may be associated with the cuticle as well.

*C. elegans *has two major sensory organs, two amphids in the head and two phasmids in the tail. In addition, there are six IL, six OL and four cephalic sensory organs in the head. All these sensory organs open to the environment so that the sensory neurons can detect stimuli. The openings of these sensory organs are also lined with cuticles that are secreted by socket and sheath support cells. Eleven *hh*-related genes are expressed in support cells suggesting that these genes products might also be components of the cuticle lining the openings of these sensory organs.

In summary, most of the *hh*-related genes are expressed in one or a few cell types that secrete cuticle. Conversely, all cells that secrete cuticle express at least some *hh*-related genes (Fig. 3 and 4, Table 2, 3, 4, 5). The Hh-related proteins have signal peptides and are therefore predicted to be secreted [11]. For two genes, WRT-5 and QUA-1, this has been confirmed [14,15]. Therefore, we propose that all other Hh-related proteins are secreted to the ECM, in particular the cuticular ECM, although some proteins, perhaps those that lack a Hog domain, can diffuse relatively freely and may not be retained within the ECM.

### Possible functions of *hh*-related genes

The *hh*-related genes encode distinct N-terminal conserved domains besides the Hog domain, and these domains only exist in nematodes so far. The structure of these domains has not been resolved and their molecular function is still unknown. However, the presumed location of the Hh-related proteins in the cuticle or the body cavity provides a chance to surmise possible functions. Considering the structure and function of the cuticle, one possibility is that the Hh-related proteins function as structural components similar to collagens. The major protein components of the *C. elegans *cuticle are small collagen-like polypeptides that are encoded by a multi-gene family with 175 members [22]. They form the basal, fiber, medial strut and internal cortical layers of the *C. elegans *cuticle, accounting for approximately 80% of the cuticle. Lipids, glycoproteins and insoluble proteins are also major components of the cuticle [23]. Disruption of collagens or the enzymes required for their biosynthesis leads to changes in the body morphology, such as Blister (Bli), Roller (Rol), Squat (Sqt), Dumpy (Dpy) and Long (Lon) phenotypes [22,24-27]. RNAi knocking-down of *hh*-related gene expression did not reveal strong phenotypes, although some disruptions of alae [9,14,15] and some low penetrance Rol were observed [14]. The RNAi defects in alae are often found in those *hh*-related genes expressed in the hypodermis or seam cells [9]. The most severe phenotype is molting defective (Mlt) [9,14,15]. However, the molting phenotype did not always correlate with our expression data, but some of our GFP reporters may not express in all tissues. Some of the RNAi Mlt phenotypes might also be the indirect consequence of partial gene inactivation, for example, in *wrt-5 *deletion mutants we observed primarily embryonic lethality but rarely Mlt [14]. In the case of the *grl *genes the large-scale RNAi screens resulted in mostly wt phenotypes.

Altogether, the functional data suggest that *hh*-related genes might not act as structural components in the cuticle or in the biosynthesis of collagens. However, there are three proteases, *nas-36*, *nas-37 *and *cpl-1*, located in the cuticle and involved in the process of molting [28-30]. These proteases are enriched in conserved cysteine residues. Their expression shows oscillation and their mutant phenotypes are Mlt [28-30]. The *hh*-related genes products also have conserved domains containing multiple cysteines, a few show cycling expression, and many have Mlt phenotypes when mutated or knocked-down by RNAi (Fig. 5 and [9,11,15]). Therefore, it is possible that the Hh-related proteins function in the same pathway as the proteases.

Another possibility is that the Hh-related proteins function as signaling molecules like Hh. Smo is a necessary component for transduction of the Hh signal. The expression analysis (Fig. 5) and the functional analysis [19-21] of the two *frizzled *genes T23D8.1 and F27E11.3A reveal that they are unlikely substitutes for Smo. Because of the lack of other pathway components between Patched and Ci, there is little doubt that there is no bona fide Hh signaling pathway in *C. elegans *[4]. However, this does not exclude the possibility that a portion of the pathway or some specific mechanism is conserved. Some of the Hh-related proteins might interact with Ptc or Ptr, since particularly the latter gene family is expanded extensively. At least some of them are involved in the same developmental processes such as molting, as shown by RNAi experiments [9]. In addition, one of the Dispatched orthologs, *che-14*, has been found to have an expression profile similar to that of some *hh*-related genes [5].

The expression data we present here suggest that many of the *hh*-related genes function in the ECM of cells in contact with the outer environment of the worm, particularly the cuticle. However, we do note that some expression is seen also in other tissues, for example neurons. Further, *wrt-5*, while it is expressed in seam cells, exerts its function already in the embryonic seam cells and mutations in *wrt-5 *lead to arrested embryonic development; this is before the cuticle is synthesized. Therefore, some of the Hh-related proteins may also be involved in other functions, perhaps to ensure the proper extracellular environment for neurite outgrowth and function, or membrane structure and fusion.

## Conclusion

We have compiled the definitive list of *hh*-related genes in *C. elegans *and *C. briggsae *and refined their ORF predictions. Further, we made a comprehensive survey of expression patterns for the *hh*-related genes and found that all of these genes are expressed in one or a few of the following cell types: hypodermis, seam cells, excretory duct and pore cells, vulval epithelial cells, rectal epithelial cells, arcade cells, pharyngeal muscle or marginal cells, support cells of sensory organs and neuronal cells. Almost all of these cells secret cuticle components. The expression levels of some *hh*-related genes can cycle in phase with the molting cycle. We propose that a number of these proteins might be components of the ECM and that they contribute to the proper function of the ECM.

## Methods

### Bioinformatics

Tblastn and blastp [31] searches of *C. elegans *and *C. briggsae *were performed at the Sanger Center ([32,33]. The genomic *C. briggsae *sequence data were produced by the Sanger Institute and the Genome Sequencing Center at Washington University Medical School [34]. As queries the previously known members of the *wrt*, *qua*, *grd *and *grl *families were used [11]. In addition, PSI-blast searches were performed at NCBI [35] in an attempt to identify additional divergent ORFs. ORFs and associated relevant information were retrieved and stored in a local database (Filemaker Pro 6, Filemaker, Inc.). Multiple sequence alignment for the different gene families was performed at the protein sequence level using ClustalX [36] and MAFFT [37,38]. Problematic regions in ORFs were identified by breaks and truncations in conserved motifs, or divergences in ORF structure between *C. elegans *and *C. briggsae *and between paralogs within gene families. In *grd *and *grl *genes the conserved domain usually lies at the C-terminus, and upstream there can be a region of variable length with repetitive amino acids (see overview in [4]). A major guide in correct identification of the N-terminus was the presence of a signal peptide for secretion. Analysis of signal sequences for protein secretion were performed at the SignalP web server [39]. If prediction errors were suspected, the genomic sequences of putative *C. elegans *and *C. briggsae *orthologs, or even closely related paralogs within *C. elegans *were compared using the dotmatrix program PPCMatrix [40]. PPCMatrix also allows comparison of a protein sequence against a genomic sequence translated in three-frames to identify conserved exons that might have been missed in automatic ORF predictions. ORFs were subsequently corrected manually. The correctness of the N-terminal exon predictions was corroborated using the SignalP server on the N-terminal sequences for each ORF. Putative cleavage sites for the signal peptide are given [see Additional file 1]. For multiple sequence alignment of the Ground and Ground-like domains MAFFT was used [see Additional file 3]; in case of GRD-1, GRD-2, and GRD-11 the four domains were extracted by hand and added to the alignment labeled with R1 to R4. Phylogenetic analysis of the alignment was done using Neighbor joining as implemented in ClustalX [36]. Bootstrap values for 100 trials are shown.

### Worm culture, microscopy and image processing

All the worms were cultured at 20°C as described [41]. Microscopy and image handling were performed as described [15]. For each strain, the embryos and all stages of larvae and adults were mounted on 2% agarose and paralyzed by NaN3 for observation. Live GFP time-lapse: for each worm strain eight transgenic larval worms were singled on NGM plates with food. The worms were observed at 1 hr intervals [15]. Each recording lasted for at least 13 hrs to observe at least two molts. To produce the intensity graph we calculated the average pixel intensity (using the histogram function in Adobe^® ^Photoshop^® ^on the raw image data) over the area of the worm (selected by magic wand and lasso), and subtracted the average pixel intensity of a background rectangle adjacent to the worm. This was done for each image frame to yield the points plotted in the graph.

### Generation of constructs and transgenic worms

The constructs of the putative promoter regions fused with GFP were made by two methods. One was to amplify the promoter-containing region of a gene by PCR using genomic DNA as template and cloning it into the promoter-less vector pPD95.67. This method was applied to *wrt-9*, *wrt-10 *and the two *frizzled *homologs. For each construct, two or three transgenic worm lines were generated. The translational GFP fusions of three genes *wrt-9*, *wrt-10 *and *grl-4 *were also made in a similar way. Both the promoter and the coding sequence without stop codon were amplified by PCR and inserted into pPD95.67. All these constructs were injected into N2 worms together with the marker *rol-6 *(pRF4).

For the high-throughput transcriptional fusions, the following, second method was used. Upstream promoter containing regions were amplified by PCR followed by fusion PCRs to a GFP-encoding cassette derived from pPD95.67 [16,42]. These PCR products (10 ng/μl) in TE buffer were co-injected into *dpy-5*(*e907*) with pCeh361 (carrying wild-type *dpy-5 *for rescuing the Dpy phenotype) as a marker. The putative promoter-containing region was chosen to be the 3 kb region upstream from the start ATG. If it was shorter than 3 kb, because of another upstream gene, then the entire intergenic sequence was taken. For each PCR construct we generated at least one and in many instances two transgenic lines. In some cases, when no GFP signal was observed, either a new construct or a new transgenic line was generated to attempt to get a GFP expressing line. A list of the primers used to PCR the upstream promoter-containing regions and of the respective sizes of the regions is given in a separate table [see Additional file 2].

## List of abbreviations used

DIC, differential interference contrast, ECM, extracellular matrix, EST, expressed sequence tag, GFP, green fluorescent protein, *grd, groundhog, grl, ground-like, hh*, *hedgehog*, Hh-N, N-terminal domain of Hh, IL, inner labial sensory organ, OL, outer labial sensory organ, OST, open-reading-frame sequence tag, *ptc, patched, ptr, patched-related, qua, quahog, wrt, warthog*.

## Authors' contributions

LH made observations of all the transgenic worms, processed all the data and wrote the manuscript. RJ was responsible for generating the constructs and transgenic worms except the two *frizzled *homologs and *wrt-9 *and *wrt-10*. GL generated the constructs and transgenic worm strains of the two *frizzled *genes and *wrt-9 *and *wrt-10*, and made observations of them. DB participated in the design of the study. TRB did the genome analysis and ORF predictions, conceived the study, and participated in its design and coordination and helped to draft the manuscript. All authors read and approved the final manuscript.

## Supplementary Material

Additional file 1 **List of *C. elegans *and *C. briggsae hh*-related genes**. The *C. briggsae *gene names are provisional. The signal sequence cleavage site predicted by the SignalP server is shown, followed by the full protein sequence. Click here for file

Additional file 2 List of primers used for generating the reporter constructs and all transgenic worm strains. Click here for file

Additional file 3 **Multiple sequence alignment of the Ground and Ground-like domains from *C. elegans *and *C. briggsae***. Sequences were aligned using MAFFT. Flanking sequences were trimmed to retain the Ground and Ground-like domains in the center. For GRD-1, GRD-2, and GRD-11, which each have four Ground domains, the Ground domains were extracted and added to the alignment. The alignment was displayed in Clustal_X, with the default color scheme, except for the cysteine residues, which were highlighted in yellow to visualize their conserved nature. Some *Brugia malayi *(*Bm*) sequences from previous work [4] were also included in the alignment. Click here for file
