# Supplementary material for: Comprehensive analysis of gene expression patterns of hedgehog-related genes
Source: BMC Genomics. 2006 Oct 31;7:280. doi: 10.1186/1471-2164-7-280 (PMC1636047; doi:10.1186/1471-2164-7-280)
Supplement: Additional file 1 — List of C. elegans and C. briggsae hh-related genes. The C. briggsae gene names are provisional. The signal sequence cleavage site predicted by the SignalP server is shown, followed by the full protein sequence. [file 1471-2164-7-280-S1.pdf]

species	gene	ORF name	type of domain	chromo-some	predicated cleavage site	protein sequence
Ce	wrt-1	ZK1290.12	wart hog	II	AAS-AS	MVMNPLTATFLAALIGTAASASCGSSGIPFRFEVLPSGQPVLGCGSPTCFGAENGGRDL<br>RHDSSFMAGADGDDGFFRDGDLARVRVRDPDAPAQMANCPREFSSSSCSNPMTWVG<br>GFKASDNGDLSLQCCHYEGLRFAQEVGRPVVHPGEVYSGGEVLRDGRQTGFDAISNV<br>RKITSGDGTVAYEVTVTRMNCLPNPGEESNEVSFDIQRDIGRILDKVGETAASGVQTNHI<br>EADQRLSPSTDVQSDSYVSPTEADPQEPVEQFVQVGEQVVPVTSAGYYYPVASGVPA<br>CFTGNSKVMTPAGEKSMADLSVGDMVMTYEYGKMTYTRVASWLHRLPDTKAAFIKLTT<br>EQGAIIDMTPQHFIYKANCVTEEMELVYAEDMTIGDCLMVKENEKLVMTTISEKSTFYET<br>GVYAPMTETGDLIVDDVYASCHNVVKANTLSHTFLNFATSVQQKMRSVLGSLEETGHLP<br>ATSEFFLNIIDVLLPHKY
Cb	wrt-1	CBP09220, CBG12986	wart hog		AAS-AS	MVMNPLTATLLAALIGTAASASCGSSGIPFRFEVLPSGQPVLGCGSPTCFGSENGGKDL<br>RHDSNFMAGPDGDDGFFREGDLARVRVRHSDAPAQMANCPREFSSSTCSNPMTWVG<br>GFKASENGDLSLQCCHYEGLRFAQEVGRPVVHPGEVYSGGEVLRDGRQTGFDAISNVK<br>KITSGDGTVAYELTVTRMNCLPNPAEDTNEVSFDIQRDIGRILEKVGETAASGVQTNQIEA<br>DQRLSPSTDVQSDSYKSDSYVAPPESDQQETVEQFVQVGEQVVPVTSAGYYYPVASG<br>VPACFTGDAKVMTPSGEKTMSELRVGDIVQTYEHGKMAYTRVASWLHRLPETKAAFIKL<br>TTDNGPEVSMTPQHFIYKADCVTEVYDLVYAEDVNVGDCVMVKKNNDKLVLTTVVNKST<br>FYETGVYAPMTETGDLIVNDIYASCHNVIKTNTLSHTFLNLATMMQQKIRSLMGLFEETG<br>HLPVTSEFFLSIIDVLLPHKY
Ce	wrt-2	F52E4.6	wart only	X	ALA-SY	MHTPIIFLLALVPVALASYCGQSAIPYTFQVLRSGYPVLGCARPKCFGWTANGTRAGETA<br>QFYRVAGKDDGYLRRSDQFIKSPSKNPNFVPQLAICTDEYKSKTCEEGEWVGGLSPQS<br>DPFADQLEMKCCSYQVLISAEDRGNAIVKQGQLVVGGEVLDGSRLVAFDYISNLSKSVS<br>ENGTVVYVASIKRMPCFDEEQAVENKKRENTIVEAAAVVEQPATTPTASSVVTHQPSVT<br>VPQQGAPLNIQPYAPQQQQVQQPAQVAYPGVVNPATNYGQFSQQAYGVAQNGQYSG<br>YPQYAQQQPQPQYYQDPFAAMLQQQHQAFQQQVYANQQQQQQMQQQQQQQQAQ<br>PQQQQQPAQPQQQLGFAPLPQFPQPMMFQAPQVVAQPGVAAAAGVPQMPIGALPQM<br>PVQMTSTLPPMTLPKLEDLPKLQIPSVEDVEQVIPPVQRAILTSVAKFFGVL
Cb	wrt-2	CBP03528, CBG14131	wart only		ALA-SY	MHTPIFYLLALLPVALASYCGQSAIPYTFQVLRSGFPVLGCARPKCFGWTANGTRAGET<br>AQFYRVAGKDDGYLRRSDQFIKNPSKNPNFVPQLAICTDEYKSNTCEEGEWVGGLSPQ<br>SDPFTDKLEMKCCSYQVLINSQDRGNAVVKQGQLVVGGEVLDDGKLVAFDYISNLSKTV<br>SKNGTVVYVATIKRMPCFDEEQTETKKQENTIVEAAAAVEPPATSTTATQQPAATVPQV<br>QGTQTLNIQPYAPQQPQALPKNSVPTATYGQFSQQAYGVAPNGQYSGYPQYAQPQAQ<br>PQQQQQQPQPQYYQDPFAAMIQQQQAALQQQQALANQQFQQQQQQQQQQMAFAP<br>LPPFPQPMMFQQPQIVQVAAPGTAGAAGATSPQVVATGVLPVQMTSTMAPMTLPKLED<br>LPKLQIPSVEDVEQVIPPVQRAILTSVAKFFGVL
Ce	wrt-3	F38E11.7	wart only	IV	SLA-DY	MLYHVEMFTIILLFGFSLADYCGSDQVPYGMEVHHSGVVRLMCSKPNCYDKNYSDCPE<br>RAESRHGCQKSNQWVGGFEKNIEGDLYTMCCEFEGLEKYAKVRYSDVRIRRGEFFEGE<br>EKENDDGDVVKFDVIKDIRMHKDDEGQAYYNLTVLSFNCESIPDVKPAWYQKSQWPYF<br>QFAKN
Cb	wrt-3	CBP05141, CBG21671	wart only		CRS-DY	MWWQAVTIILVAFAIICRSDYCGSDQIAYGMEVHHSGVIRLLCSKPNCFDKNYSDCPERA<br>ESPQGCKKSNDWVGGFDKNIEGDLSVMCCEYEGLEKYAKIRYTDVRIRRGEFFEGEEK<br>ENADGDVIKFDVIKDIRMHRDSDGHAYYNLTVLSFDCESIPDVKPAWFQKSQWPYFQYK<br>K
Ce	wrt-4	ZK678.5	wart hog	X	TYG-SE	MRFSLLALVLLSSSYKFTYGSECGDSTIPYSLEVLSSGQPILGCARPTCFGWHSNGHQL<br>PTNAKFFRIDQQSDGFLRDDPLAIHTFDAADPRVYAQQQASCEQEFQSLSCNPEDQWV<br>GGIAPVMNASTTKIVAYKCCTYAPLRASIDRGVATVSGGQIVVGGEIFADNKPYAFDYISN<br>VEKKIDSEGEIFYEVNIKRFSCLDLQKVDRSVPEILNSENTIRHVNGHRFVVHQAPTVDVE<br>TPVETGQLVVPQGVQNGQEVIIEEIVAQEGFVQETNPQPPPPPGQQGGFVQPQGFQPQ<br>GGFQPQGFQPQGFQPQAFQPQVVQNPVPAAPAGYAPMGFAPSGLQLYYCFPGDAMV<br>NVYNGGFKRMDELAVGDWVQALDKNGSQVTFIPVQYWLHRDPKQVADFVEFTLDNGE<br>TFSLTEKHLVFVTQCSVPYSEDENINANPVPAERVNIGDCFYIAHRKKSQMYQRVKVLDI<br>NIVQKTGIYSPMTSRGHLLVDRIHASCHSETDNYSLQNTFFTNVLRWKSQIRNYFWTVED<br>STNEDNIGYGLNGVMAVLDIVIPSKLM
Cb	wrt-4	CBP15980, CBG07767	wart hog		AFG-SE	MRSLIWIVLLSISYEAAFGSECGESTIPYSLEVLPSGQPILGCARPTCFGWHPNGHQLPTT<br>AKFFRINQQSDGFLRDDPLAIHTFDAADPRVYSQQHATCEHEFQSFSCNQEDQWVGGI<br>SPVMNATNTQVIAYQCCTYPPLRASTDRGLATVSGGQIVVGGEVTENNKQYAFDYISNV<br>EKKLSAEGEVFYEVNIRRFSCLDLQKADRSVQEILNAENIIRQVNNQKAVAHQAPIAAVDT<br>PIEAGELVVPEGVVNGQAVIIEEIVAQEGFIEEVTTPVPPPPFQPQGFQQPPPQPFQPQPI<br>PQGFQQPVQPQVFQPVPVQQPVPVPAQPAGYFPYFSAPAGLELYYCFPADATVHVYD<br>QGTKRMDELEVGDWVQAFEKNGETVTHVPVQYWIHRDQTQKATFIEFSLDNGEKFSLT<br>EKHMVYVSQCENNNYVEGINSNAVPAEKVNVGDCFYVAHRTNSKLYQHVKVLDINKVK<br>KTGIYAPMTSVGHLLVNRIHTSCHSETDNHTLQDTFFANALHFKNLLMKFFGTADSTKEE<br>NLGYGIHSLLDVVDLVLPAKFV
Ce	wrt-5	W03D2.5	wart only	IV	TLA-DY	MCSMWLMASWLMAFVAGSTLADYCGDHKVPFGMEVHKNGNVNILCSRPSCHEKKYAE<br>CPERATSTTCSTNSSWVGGVTQHSDGSLRLMCCEYDLLPTYSTIQYEKLTIRTGEYFEG<br>DKQMEGDVVTAFDLIGNIEQVKEPDGKYSYNLLIYRYHCGNIPDTPPAWYMKKQWPYW<br>EK
Cb	wrt-5	CBP11846, CBG21493	wart only		ILG-DY	MSNLHKLLYLLLPTVILGDYCGEHKVPFGMEVHKNGNVNILCSRPNCHEKKYAECPERA<br>MSSACTTNSSWVGGVTQHVDGSLKLMCCEYDLLPIYSTVQYEKLQIRPGEYFEGDEQM<br>DGDTVTAFDLIGDIQQVRDAASGNFTYNLLIYRYHCGKIPDSPPAWYMKKQWPYWTPVA
Ce	wrt-6	ZK377.1	wart hog	X	VLA-DS	MTLLNLFYCFCLLFGAVLADSIHDGGSCGTNSIPYKMEVDSEGKPVISCEAPSCLGVSSS<br>AARRPRVLDVSCDPFKEIVCVKDLQWTSGLVEINNGTHRTLKTECCSYEGMSDAKTIKSI<br>FLGPGQSFVGGMVEKDGEQSGFDLIKEIRKTVNADNQVQYIVGVYRMPCEATSDSSEE<br>ALPLLSRNRRKLRDRVGKYDDYEEDRNYRSERRRPFAMRRRALLQRLEDMYDDYDYEF<br>RVVRRPFRKSRLPYNENALWPLQYSSPQRSRTFADNTYNKETGLLTTMQDGTAALSNY<br>GDSSVESGPLPPPPSSNYIDSQNVAPASPVVQSPAYPQTPAEMPLPPQSGSYSGSYSG<br>YPTADASQYNAYPAMQQPAYQPAYQPAYQPAYQPAYQPAYSARGYSPNLNGLF<br>GGTGMQCFSGDMEVETEDGIKMIKDLKIGDKVLSMDEAFVTYSPVIMFLHKRDEEIAEFN<br>LIETANGHSIKLTDNHLIYVSDCRTRSDLKLVAAKEVKMDDCIHVTTDSNVVIKKKVSKISK<br>VIETGIYSPLTSTGDIIVNRVLASCHSNLALKSLQQTFFSLYKRTSSVFHNLMFFKSSTEEG<br>DLPVGVETLTSVMDLFIPQSFV
Cb	wrt-6	CBP09579, CBG14222	wart hog		AIA-DS	MRLLLNLCLPFCILFASAIADSYHDGGSCGTNSIPYKMEVDSDGKPVISCEAPSCLGLTSS<br>LKSRPRELSVSCDPFKEVVCVDELQWTSGLVEINNGTHRTLKTECCSYEEMATAKNVKS<br>IFLGPGQSYVGGLVEKDGEESGFDLIKEIRKTVNADNQVQYIVGVYRMVCNARSDSSEE<br>LPVLSRNRRKLRERVGKYDDYEEDRHYRRRRPFAMRRRALLQRLEDYYDDYEYDFRP<br>MRRPFRKSRLPYNENALWPLQYSAPQRSRVFAENTYNEKTGLLETMQDGTAALSNYGD<br>SSVESGPLPPPPSSSYIDSQNIAPASPSVQSPAYPQNSQMPQPPPSDSYAGSYQQQNS<br>YTSYNGYPTADNSQYNGYPAMQQPAYQPAYQPAYQPAYQPAYSPSSYSGYSPNLNGY<br>FTKMQCFSGDMEVETEDGVKLIKDLKIGDKVLSMDEAFVTYSPIIMFLHKRDDEKAEFNLI<br>ETSNGHSIKLTDNHLIYVSDCNARSDLKLVAAKEVKMDDCIHVTTENNAVIKKKVSKISKV<br>VDTGIYSPLTSTGDIIVNRVLASCHSNLALKSLQQTFFSLYKRTSGVFNSFALFKTSQDDG<br>SLPVGVETLTSVMDLFIPQSFV

Ce	wrt-7	ZK1037.10	wart hog	V	SFG-SS	MNISKCVLVVALLSLCCKLSFGSSCGETTIPFSFEILPTGQPVLGCARPTCFGWDPKGYH<br>LPTDARFVRIDRKRDGFLRDDPIYTYPFTPDGSKMYLQQNSTCEPAFQSAMCDSKIQWV<br>GGVEPVQDVNSTHDIAYQCCTYPPLRESTDRGMTLVAAGQIVIGGEVFKNGSQYAFDYI<br>SNIAKNIDEYGKIFYEVNVRRLACLDPHNADRSVDEIWDSENTIRKVNGKKAMAHQVPNV<br>AVGNAVPQYNVYPAVAQQPAPYSYLPAAPQCQQYYCFPNDAVVNVYEKAVKRMDELEI<br>GDWVEALDENGEDITFLPVKYWLHRDPEQEAEFLEFSLDNGETFTLTEKHLVYTTECRQ<br>NSSELKISWESISAGKVNAGDCFYLAQSEALTKYRLVEILDIKRVKKTGIYAPMTSQGHLL<br>VNKIHTSCHSEVDHHILQNSFFKHVLKWKNKITKYFWSYETERNIGQSLNSLIAIFNLVVP<br>SNMY
Ce	wrt-8	C29F3.2	wart hog	V	VFG-SR	MNYLLLVSGLLSVWQPVFGSRCGESTIPFSLEILPSGHPVLGCARPTCFGWHPKGYQLP<br>TTAKFSRLNRKLDGFLRDDSLFTYPFETDSSKIYKVQNSTCEPGFQSSKCDSKDQWVG<br>GIEPETDAFQDVAYQCCTYAPLRESTDRNIATVSAGEIVIGGEVYQNESQYAFDYISNIEK<br>SMDENGEVYYEVNIRRFACLDPHNADRRIDEVWSSENTIRKVNGQKPIAQQAPNVAVNA<br>PIEAGTFDGEVVDGQTVVIEEIIAQQGFIVENETTVAPFAGPFQAQGFQPRFGAPQGFQP<br>AFQQPPPQQFFPQNFQPVVQQPVQFPAQPVGYAPYAPAGWQLHYCFPADAEVNVYEK<br>GVKRMDELEVGDWVQALHGKETTYSPVKYWLHRDPEQEAEFVEFLLENGESFTLTEKH<br>LVFATDCQQNVKNLDDLNPTSTGKINIGECFFMAQPENASKFQKVQILDIQRVRKTGIYA<br>PMTSLGHLLVNQIHTSCHSEIDHHLLQNSFFKHVLKLKNRISKYFWNEESNTEGNIGTSL<br>NFLIEIFELIVPSKMISY
Cb	wrt-8	CBP15980,<br>CBG07767	wart hog		AFG-SE	MRSLIWIVLLSISYEAAFGSECGESTIPYSLEVLPSGQPILGCARPTCFGWHPNGHQLPTT<br>AKFFRINQQSDGFLRDDPLAIHTFDAADPRVYSQQHATCEHEFQSFSCNQEDQWVGGI<br>SPVMNATNTQVIAYQCCTYPPLRASTDRGLATVSGGQIVVGGEVTENNKQYAFDYISNV<br>EKKLSAEGEVFYEVNIRRFSCLDLQKADRSVQEILNAENIIRQVNNQKAVAHQAPIAAVDT<br>PIEAGELVVPEGVVNGQAVIIEEIVAQEGFIEEVTTPVPPPPFQPQGFQQPPPQPFQPQPI<br>PQGFQQPVQPQVFQPVPVQQPVPVPAQPAGYFPYFSAPAGLELYYCFPADATVHVYD<br>QGTKRMDELEVGDWVQAFEKNGETVTHVPVQYWIHRDQTQKATFIEFSLDNGEKFSLT<br>EKHMVYVSQCENNNYVEGINSNAVPAEKVNVGDCFYVAHRTNSKLYQHVKVLDINKVK<br>KTGIYAPMTSVGHLLVNRIHTSCHSETDNHTLQDTFFANALHFKNLLMKFFGTADSTKEE<br>NLGYGIHSLLDVVDLVLPAKFV
Ce	wrt-9	H02F09.1,B03<br>44.2	wart only	X	GAS-YC or<br>SYC-GS	MRHRAFSFQIALVLALLQPFGASYCGSNGVPYSLEILSDGSPVLGCAQPTCMAEPQEDE<br>EDSVFIANTAGGQEDGFFREGDRQKKSYTQSYKPKAECPGEFSDFACTKKNQWVGGIDFI<br>DHPRQPLVLQCCTFEGLRFSQDVGVTTISAGEAVTGGEVVRDNRQISFDVIANVRKLVD<br>PDDPKRTYFEVTVRRMNCLPDPPEFEVAYDDDVEPEIQRVLGNATNSAMNMGHENHPH<br>MAQEKKVSKNRPYKRKPKTSSTNNVVSPFVEHHEGEKKSREPFIGSFGATIFNPTAENN<br>KEQETPRAPPRRVHQPYTRRILTPKPRIVHTTTVMPTTHAPPVQVQVEPVTQPTPPPNP<br>FVFAPLPPFPQFGLPQPNLFQFPAPPAPPPFGVPQAPQLAPLPQHQHQFGFFQPPPQP<br>QPQIFGGYGLNNGGGYQQPSYGLQGVQTVQQNQLPEMDVYTRSLLTNPNSPFALQLP<br>TFGAAPPAPQFFAPQQQIQDQQKNLQRVNIQAPQREQSNQPAQLMLQPQPDPNQAPN<br>HDTLGALYRPPPFAALGTTLYNFQSHNG
Cb	wrt-9	CBP18687,<br>CBG16423	wart only		IET-SY	MRHGAFILSSFLLLLQLYVIETSYCGENGVPYSLEILSDGSPVLGCAQPTCMSEPLEDNE<br>DSIFIANAAGQEDGYFREGDRQRRSYSQSYKPKAECPGHFSEFACTKKNQWVGGIDFID<br>HPRQPLVLQCCTFEGLRFSQDVGVTTISSGEAVTGGEVVRDGRQISFDVIANARKLVDP<br>DDPKRTYYEVTVRRMNCLPDPPEFEVAYDDDVESEIRRVLGNATNSAMNIGHDNHPHIA<br>REKKVPINRPYKRKPKTSSTHNVVSPFVDGHDGEKKPRSEPFIGSFGATIFDPTAEHNAQ<br>EESAKSATPPPKREHVPYTRRILTPKPRIIPTTTQTPTTIAPPVQVQVEPVSPMPAAPLNP<br>FAFAPLPPFPQFGLPQPNLFQFPAAPPAPAPLPPAPGPTISGVLQAPQLTPLQPYNHQFA<br>FFQPQPPIGQYGLNGFHQPAPVYGLHGVQNVPSDQFNNLDVYTKSLLTNPQSPFALQM<br>PTFGAAPPAPQFFPQQQPTIEQNASLQRIDIQAPQREKIAQTAAQFSLQPQAANQIPKKD<br>TLGALYRPPPFAALGTTLYNFQAHNGK
Ce	wrt-10	ZK1290.8	wart only	II	VLA-KD	MLLVSVISCLLISVLAKDAVTPRVGSQCTKNQVVRKLTVYEDGALEAECGPVPCGEVGK<br>RCIDDQTSCRAETDVFSGMRWAPNGESILLRCCTMHAKNKIYVGTDVVAAGSFYEGGE<br>VAEKDLYGDKGGAEYDFVANARTEQGGVRVWVYRMICAKGEKPVDFDPITTTSAPRVIK<br>TTPAPTTTPTVEEEAEEAEQLEEDQPNDNEAEIVESNDEEEVVEEETEEEEEVTTTPAPK<br>PFNPLRYRPPHFPRQSTGIRRA
Cb	wrt-10	CBP09221,<br>CBG12987	wart only		VLA-KD	MLLISIVSCLFITVLAKDANVRMGSQCSKNQVVRKLTVYEDGALEAECGPVPCGEVGRR<br>CIDDQTSCRAETDVFSGMRWAPNGESILLRCCTMQAKNKIYVGTDVVSAGSFYEGGEV<br>AEKDLYGDKGGAEYDFVANARTEQGGVRVWVYRMICAKGEKPVDFEPITTTAVPKVVK<br>TTTPEPTTPVVEEEEDENEQLEGEEQTNENVAEVVESNDGEEEVEEEDEEEEVTTTPEP<br>KAFNPLRYRPPHFPRQSTGVRRS
Ce	grd-1	R08B4.1	ground hog<br>4rp	X	SEA-NK	MNLLIFLLCYFLGSPHYSEANKIKLKSSGRSADINHDSPCIWNKTQSWVHPYQSITVECC<br>DEELSKLIHKTILDAGNNAKLGNLAKFIQRRSQLFYHMSFETIVSRENFAISTHYHGTHSC<br>RVHDNNLYYLVYETPVQYDPFNMKTEDYLASIDSADPLGSTKPANLRGDFPDVREDSTA<br>GEDLSVQPSIGNILQYPNKFAWPDPDEEVSKKIMLAEQDAIRSINELMESVTEISVMNSG<br>AVVNSTDSLNITEIELPNPTPIPRNFANLREKDRLPENTHCDKERKDGNRCCDGRLASTM<br>RDAMRQMATSPDFGQGKEGIIAAELQQKVQQRFQKSYEIIVSQSDFVISTYTAGDNFCKF<br>DNKGFYILAYVSPKQYDIDEKEDEMKLAATSNKDPLGANTTMFENEAPWHVALKLDTYG<br>DRAGYPVGSHCTQARTGSKCCSLILFNAMKSGYDSHVATSNFDPYDIRNISKAVQWSVE<br>EILQHSAEVIVSLDDFAYATYNNNSYTCKYRVDKYHILAYTTPNHDLDNYDEMATSIIDSE<br>PEAQIVYPAVDRTANQIPFQNTPSPYWTTPVPIPTQVYMPMQYTQQPMQPMYNQQPFF<br>NQQPMFNQQPMYLQPAFNQAPLPYQYPAQPSQFNPFLASFRRFKRQIGRTPYPIHQNV<br>YNDIGSAKPFNCPADLSGLSGMACCDGGLQFEANKVIDQAKQEPDFDKHNTRNLAKLM<br>TRAVQKRFGTTFESVVAEADFSWGTNKFNGRTCKIDSQGYNALTYQSSSKPPPPSDFID<br>IPNDPTLGGPTGSSGGGGGGGNGGSGGGGGGGGGGSGGSGGGGSNSNSGGGGGN<br>GGGGNGGGGNGNGGGAGDGNGGAGAGNGNGAGAGNGNGAGAGNGNGAGAGNGN<br>GAGAGNGNGAGAGDASAAAAAAQAQAAAAAAQAQAAAAAAAAQQAAAAAAAAAANAQQAA<br>AAAAAAANPLSALVAATGACFSLDTWVTTPTGKKRMDQIDIGDYVLTADLEKTYFTPITL<br>WIHREPEKVQEFLTIMTEYGKTLRITSRHFMYRNKCGKSYPQYIKMLPHDGEAIFASDLE<br>VGDCVVVLYKGKYRQQKIETITRSVRTGIYSPLTNNGRIIVNDMLASCYSEIQQNTLQTTFF
Cb	grd-1	CBP10540,<br>CBG17433	ground hog<br>4rp		VDA-EE	MNLPIFLLCYFWGSPLFVDAEEELKYKTFGTSTDTKHQSPCIWNQTQSWIHPYQSITVEC<br>CDEELGDLIRKTINDAGRNAKLGNLAKFIQRRAQLHYHISFESIISKSNFAISTHYHGTHSC<br>RVHDNNHYYLVYETPIQYDPFNMRTEDYLSSIDSADPLGSTKPPNLRGDFPDIREDSTAG<br>EDLSIQPPIDSILQYPNKFAWPDPDEEISKKIMKAEEEAIQSMNELMESVTEISIMNSMVNA<br>TDGLNITEIELPNPSPIPRNFANLREKDRLPENTHCDKEQKDGNKCCDGRLASTMRDAM<br>RHMATSPDFGKGKEGIIASELQQKVQQRFKKSYEVIVSRSDFVVSSYNGGDTFCKFENK<br>GFYILAYSTPKQYDIDEKEDEMELAATSNKEPLGSNETMFENEAPWHIPLQLERSGERA<br>GYPVGSHCTEARTGSKCCSLILFNAMKTGYDNHVATSNFDAYDIRNISKAVQWNVEEVL<br>QHSAEVIVSLDDFAYATYNNNSYICKYRVDKYYMLAYTTPNHDLDDYDEMALTKIETEPE<br>AQLVFPSVDRSTNQVPFQNTPAPYWTTPRPIPTQVFQPIQYTQQPMYHQQQQQPVYNQ<br>QQQFFNQQPMYHLQPSFNQAPLPYHYPGQASQFNPFLANSFHRSKRQIGASPYPIHQN<br>VYNDIGSAKPFNCPAGLSGLSGMACCDGGLQFEANKVIDQAKQAPDFDKHNTRNLAKL<br>MTRAVQKRFGTTFESIVAEADFSWGTNKFNGRTCKIDNDGYTALTYQSSSKPPPPSDFL<br>DIPGDPTLGGPTGSSGGGGGGGAGGNGGGGGGGSGGSGGAASADASSGGGGNGG<br>GGNGGGGNGGGGNGGAAGNGNGAAGGNGNGAAAGNGNGGGAGNGNGAGAGNGN<br>GAGAGDASAAAAAAHAQAAAAAQAQAAASAAAQQAAAAAAAANAAQQAAAAAAAAANSN<br>DAFSALAAAAGGACFSLDTWVTTPSGKKRMDQIDIGDYVLTAGLDETYFTPITLWIHREP<br>ERVQEFLTIMTEYGKTLRMTARHFMYRNKCGNSYQKYIKILPHDAEAIFAEDLRVGDCVV<br>VMYRGKFHQQKIESIIKNVRTGIYSPLTNNGRIIVNDMLSSCYSEVQQNTLQTTFFWAYDK

Ce	grd-2	F46B3.5	ground hog 4rp	V	VDA-VC	MKFPTFLLFYFTGFLGGYQVDAVCIWNRTQSWTVPYQSILVECCDKPLKQLILDTIHGTN<br>FESLANGDFAKIVQRRAQMEFHSSFETIVSKTNFAISSHYHGPHTCRVHKKGHYLLVYET<br>PVQYDPYNLPIENYLASIDSSDPLGSTKPFGLRGDFPDISDDPTAGDDLSIQPPSDQILQF<br>PNKFAWPDPDDGTHEPSNKIKRADFDEEVDDLMEISVMDSNSNSTHLEEDLKKEFASKF<br>NTDLDRPGPQPVPRNITNWRKKERYPKGTHCESGKRDGNKCCDARLAATMRDAMRQ<br>MATSPEFGPGKEGIVAAELQQSVQTRFKKSYEIIVSRSDFVATTYTAGEKFCKFKTKNFVI<br>LAYVTPRQYDIDKKDDERILADVSNRDPLGSSETKFPDEEPFHKELQLEESGDRAGYPV<br>GSHCSNDTRTGSKCCNLRLFNAMKDGYDRHKASPNFDRYNIRNISKAIQWSVEEVFQH<br>SAEVFVSLDDFAYATNEKNEESCKYRVDKYYVYVYTTPVPYPIHQPTYNDIGSAKPFHCP<br>PDLSGLDGTACCNSGLQYEINRILDEARYSDSYHKHDTRYLAKMITRAVQRRFNTTFETI<br>VAEADFSWSTNKFTGRMCKIDHEGYNALTYQSSLRPPRAIDFIDFPNDVTLDGPTFVEN<br>GWFGNGNGALSAQAQVVVQAQIAAQAVAPPQPPVFVQQFVAPQFVQFVAPQPQFVAV<br>APQPQFVAVAPVPPPVAPAVAFAGGGGGGGGCFSSDTLVTTPSGKKRMDEIDVGDYVL<br>TANRVKTHFTPVTLWIHRESEKLEEFLTITTERGSTLQLTPLHFMYRTKCNESSEFLKILP<br>ENHEAILASYLEIGDCVILTENTKFRQEKINQTTRGLKTGIYSPLTKNGRIIVNDMLASCYS<br>EVQANVLQTTYFWVFNRLRQKVLNLFGILHMNEIELPTGTAVYKELLSLVIPMGK
Ce	grd-3	W05E7.1	ground only	IV	VRA-QS	MKFLLLLGAVIAVRAQSDTCPSLKDRQLAKPGQLFCCDSTIKTVVETGMQTLDLFGAGG<br>PQTLGPIVQALSTFVQQHFKTAYEIVMAPKDFVLNTHYNGTSLCKFQSNSYTLAIYETPAK<br>YDINSSREKYFNKFALNDKLRLPSVSKHLKQFSGLAN
Ce	grd-4	T01B10.1	ground only	X	VQG-QD	MKLILLLIGFAAVAVQGQDDNCPSMKSRKHAKSGDLYCCDSTIKTVIKRGIRTLSYFGDD<br>GPQTLGPIVQGLSTYVQRHYGVAYEIVLAPKGFILNSNYNGSSVCKFETNSYTMAVYETP<br>EHYDVNGPGEAYYYHFAANDKLRIPSVSSHLKKFSGLVNTVTERDVLRN
Cb	grd-4	CBP05429,<br>CBG22788	ground only		IQG-QG	MKFLLVLIGCIFVAIQGQGDTCPSWKDRQHAKPGKLFCCDSTIKTVVETGMKTLDLFGAG<br>GPQTLGPIVQSLSSFIQRHFKVAYEVVMAPKEFVLNTNYNGTKLCKFQSNSYTLAVYETP<br>EYYDINGPGEAYFYNFAANDKLNIPSVSEHLKSFGGLANSASGGLTGSASSLAGSLGG
Ce	grd-5	F41E6.2	ground only	V	CFA-QD	MRSLIVLAALAVTVCFAQDNCYINDSGFTCCNKELESAMKGAMGGDDLLGSADSIQKGA<br>EGSLGGKFETVVALDDFAYKSHFKEGKSCKIEKNGQYALAWQP
Cb	grd-5	CBP00356,<br>CBG01473	ground only		AFA-QD	MRSLIVLAALAVTVAFAQDNCYINDSGFTCCNKELEASMKGAMGGGDLLGSADAIQKGA<br>EGSLGGKFETVVALDDFAYKSHFKEGKSCKIEKNGQYALAWQP
Ce	grd-6	T18H9.1	ground only	V	VYG-QQ	MPSIRYRLVALVVFISSVYGQQEAAVVQTPPVQPTPNLIRFGSGEPVRAIRPNQFQIQLP<br>DIYDPLRDERPKRNNHAGYIRHPTFQVPQQQVQQFRPAGNVYIAQPNPRVVSQYPFRS<br>APVVRPYVIPQQQFQRQGQSQYIQQQYRPQQQQYSQYPRFDLRPQPVRPVRPVYIAST<br>PASRLSYTERPQTSYGDEIEDTNYLSGRLTPGPIAPVTQGYSERPPATVAPYIERPVPAR<br>PTPYIERPVPARPAPYIERPEPARPAPYIERPVPARPAPYIEPTPARPAPYIEPSTAKPQP<br>RPQPPRTRPYVAPSTTTQRYIEPTTQRPTTRRATTKRITTTTTAAPTTPRLTTARATTPLA<br>TTSRPTTPSPTTPRATTPLATTPLATTRAPLPPSPPPRTSKRPVTQAPTTPRATTTRRPT<br>TTTPRPTPRRTRRPKTTTAAPTTTTEEVTQGYEEEAVELPKYDETFVGQYYYGRRGEG<br>GNNTFPLPSCFYNPSGYVCCNLMLNELMSTSFEEVKVATNLCNVHKFATKLQKHSEKIF<br>STQFETIVSYQDFSQKIHFKKDLVCKIEVEGRFILAYATPEDVEQEKIIPTVPSQDVQKDSD<br>VLKQEVKSKIRQIEREL
Cb	grd-6	CBP11104,<br>CBG19232	ground only		IYG-QQ	MSPIRYALVALLTFTASIYGQQDFSTQVQPNNAQPEIIRFGSGEPVRAIKPNQFQIELPNIY<br>DPLREDRPKRNNNHAGYIRHPTFQVPPQQPIQQFRPAGNVYIAQPNPAVVRQYPFRPA<br>PVFRQYVIPQQQQQFQPQFQHQGQPQYVSQQKFRPQQPQQYIQYPRFDLRPQPVRPV<br>RPVYIQSTPAPSRPFYTERPQAAYGDEIEDSNYLNSRITPGPVAPKYSERPPAPQGYSE<br>RPPVPQTYSERPPVTQGYSERPYSERPATPQPYIEKPVPARPAPYIEPTPVRPAPYVEP<br>TTARPQPRPQPPRTRPYVVPTTRRPAPPRPAPTAAAYIEPTTTTPRPTTRRATVSRLSDP<br>PTKRITTTTAAPTTTTPAPTTPAPTTTPRTTPRTTPRTTPRTTTPRPTTRAPLPPSPPPRT<br>TRRTTTTQATTTPRPTTTPRPTTTTPRPTPPRTTRRRTTTTPRPTTTTAAPTTTTEVTQG<br>YEEAVETPQYDETFVGQYYYGRRGEGGNNTFPLPSCFYNPSGYVCCNLMLNELMTTSF<br>EEVRVATNLCNVHRFAIKLQKHSEKIFDTQFETIVSYEDFSQKIHFKKDLVCKVEIEGRFIL<br>AYATPEDVEQEKIIPTVPSKEIQKDSFILKEEVKAKIRQIEREL
Ce	grd-7	F46H5.6	ground only	X	ATA-AE	MMPSFVFIFLLVSSQLATAAEEYKSQTTYPESAPAATTPQPPPPPPPPKPAPYVEQSAQ<br>PQQTAPPPPPAPYPQQAVPAPAPPPAPYPQHAVPAPAPPLASYPQNAVPVPAPPPAPY<br>PQHAVPAPAPPPAPYPQHAVPAPAPYQQQPPPPPPPPHYPPPPPHYPPPPPAPHSAYI<br>DHSAPRPAYIEHSAPPPQPQYPQQQPPQYPQQRQQHSYNAGPRTYHEEPQPYPAKFE<br>YVDYPAPTTRRPYPYPSFEVLPHHEEYEPRSTRRPTTTEEPTTTSRKPRPGWKGDTEY<br>NYPERNLPLKGCFYNNHGYACCNLKLQNKMEELADELLNNGTFHRCNVQKLANDLQDK<br>VESAFKEDFETVVGLSDFAERIHFREHYVCKIEVNGRYMLAWATPDDIGTRRKRGANST<br>SDDIHEYNF
Cb	grd-7	CBP17047,<br>CBG11008	ground only		VLA-AE	MVPKSVFILLLTGVQLVLAAEEYKSQTTYPESAPPAATTPAPPPPPPPPKPYVEHSAPPP<br>PPAPAPSLAPYPQQAPPPPPPTAAPYPQQAPTQAPYPQHAVPAPAPYHEQPPPPPPPA<br>HYPPPPHQYPPPPHPAPHPAYIDHSAPRPAYPEQAPPPQPYPQQQHTYNGGPRTYHE<br>QPPPAYPSKFQYVDYPAPTTRRPYPYPSFEVLPHHEEYQPKPTRRPTTTEKSTTTHKPR<br>PGWGDTEYNYPERQLPLKGCFYNNHGYACCNLRLQNKMEELADQLLNNGTFHRCNVQ<br>KLANDLQDTVETAFNEDFETIVGLSDFAERIHFREHYVCKIEVAGRYMLAWATPDDHPAR<br>NKRGVNSTTPVEIHEYNF
Ce	grd-8	C37C3.4	ground only	V	FPA-QR or 22 and 23	MKSLRIIFCLTLLIASFPAQRSRRKQKFVRLPSGFTFPADAASNFQRDAYIPATFAPPSEKI<br>LQAPPRYLTGEHNPAYAHNTDVQGMNYAEYKQAMAPQPHPVDAYSPPPPAPMVPPVT<br>VVEPPAMPYEMTTIASVGPLTTPASVGLKKGNIGGIAQNLNDRYTSLTPEAQRAQKGHT<br>YTALGGGQFYQSLLGGKGGPGGFSPLSFFLNGGLGGTGGGGNNGFFVPVPVVIPPPPP<br>PPPGPNCFTNPSGFLCCNVTLEKTMEDAYLAAKADGASLCNVQKMATAVQAQAEKKFG<br>TTFESVAAHSDFVAKINFAGDLNCKIEIDGKFILAYATPIAEQEVNIVDASSFFSGAADKDL<br>DGVNGTKPTYIVYGPIK
Cb	grd-8	CBP11554,<br>CBG20565	ground only		SIA-QR	MFLLKVIVLLSLLNSSIAQRSRRKQKFVRLPSGFTFPADAASNFQRDSYIPATFAPPNEKIL<br>QAPPRYVTGENYLSSGSPSPTSEPHGMDYTEYKKAMQAYASTPVVTPHPPESPSIYQA<br>SPPPAATAPTIPPVTVVESPGSEIATTIAPGPATTAATMGLKKENIGGIAQNLNNRFSSLSS<br>EAKQSQRGHTYTALGGGQFYQSLLGGKGSFSPLSFFLNGGLGGGHNNGFFVPVPVVIP<br>PPPPPPPGPKCFTNPSGFLCCNVTLEKTMEEAYIAARDGGASSCNVQKMASAVQAVAE<br>KKFGTTFESVAAHKDFVAKINFAGDLNCKIEIDGKFIMAYATPMSEQEVNIVDASSFFSGA<br>ADPDLEGVNGTKPTYIVYGPIK
Ce	grd-9	C04E6.6	ground only	V	AEG-NC	MILPLLLSLFIFLPDVAEGNCHDHRYGVPSKSCKRVKDPTFQIRGDIRIVPMWEFILMKEP<br>SEKRVKRSTSFRNGTLRSVHSGGNHGLPVDSLPTSNKNLNQANSFIKTPVLHQLLIQPID<br>SSAPASPVQRHIIQSQGKPTIGQLPIHVNKSKINTNHKTKRQGVYSALAQPPPAAKWGNN<br>RPSYQNPTPSYQQGYPQQFPAPQRQQQYQPQPQTTNYNQAPQRPLYIPPPPPKLQVY<br>PPQQLAENPYQTRNGVNNYRSRNSYGTNNRKHGRNGKKGKSSKMCKMCRKLSEEDE<br>SEEKEMACDLCSKSRRKGKKKGKSLRDGMVGGRRKSSKSSEDSDDHDDSMEGEGGG<br>TENFTNDDENGADDYEDDGESTTIKPNAVSQPTILDFVERSKQQNKLMRIPVYRGKKLE<br>AESNGTSGAYEDGDDNGEHNREEEEESSQYSSNIRDMEQPTKYSSKPNVKYSYPPKDT<br>LPLQTCFHNPSGYVCCNLELNNVVESTYKEVRELPNFNPCNLQLIANKVQRASERMFGH<br>PFESIVSHADFAQNINFSGDLVCKLEIDGKYMIAYGTPYHADDAVGPQGPDGKPLPVRSL<br>KL

Cb	grd-9	CBP16567, CBG09297	ground only		VES-NC	MILSLLLSLFIFLPDLVESNCHDNRYGVPNNSCKRIKDPKFQIRGQIKIVPMWEFILMNEPT<br>GKRVKRASTTHQNRTIHAAHSGGKTFLQPLPLPQRWIPNASHNPPPRHHTLIQPVDSNA<br>PRLPPPQRHVVERQVVPILNPAQIYNANPYSNQPILAQNSKQNYSAYPPPAPPPRNPLP<br>NLYHQNLQQNATGQPAQYQQQSVNTNYNQPPQRRPPQLQVSPPNQAIETNYYTDRNV<br>GKYQSPKNSGRNRNNRRKGKKKKGRANSKMCRLCREMSEEEDSEEKEVACDLCSKSS<br>NRGRGRKKGRKQKTRTKDSSEEDDSDRDEDSPEGEGAQIDDITEDGDGDYYDDEDRT<br>TQKPTTVSQPTIIDFAKRAEQNKLMRIPVYRGKKLENKDAYRTGSGEHGDGDGHREEEE<br>KSEYSSNIREGEKQTKYSSKPNVKYSYPPKDTLPLQTCFHNPSGYVCCNLELNNVVEST<br>YKEVKELPNFNPCNLQLIANKLQRATEKMFGHPFESVVSHADFAQNINFSGDLVCKLEID<br>GKYMIVYGTPYHADDAVGPQGPDGKPLPVRSLKL
Ce	grd-10	F09D12.1	ground only	IV	CFA-QD	MRSLVVLAALAVTVCFAQDNCYINEGGFTCCNKELESVMKSSLGGSDLVGSAGDIQKGA<br>EGSLGGKFETVVAHDDFAYKSHFQEGKSCKVEKDGQYALAWQP
Ce	grd-11	K02E2.2	ground hog 4rp	V	VEA-GS	MIFLLFTLSILSHVEAGSFKNIGLSTDNKHQSPCIWNQTHAWSHPYQSLIVECCDESLKDL<br>LQNFIRQGKAGSLGDLAKVIQRKAQLEFHASFEAIICKSNFAISTHYHGSQACKVHVENQ<br>YFLIYETPVQYDPFNMHMENYLSSIDSSDPLGSTKPQGLRGGFKDHRDDVTAGENLSVQ<br>PDADNILQYPNRWSQQDAKEEIFTTEASIENAFVLGTNMSHLPDIAEIDLVGPVPVPKNFT<br>NLRIRDRLPENTHCEKERRDGNKCCSGPLASTMRDAMSQMASSPDFGPGGEGIIANRI<br>QHSVQHRFKSSYEVIVSRSDFMISTRSGEKICKFQSKGFYILAYATPKQYDIEKVEEERKL<br>ADISILDPLGSMESNFPHEAPQNVQLLLESSGDRVGFPVGSHCLEEVRTGSKCCSIDMF<br>NAMNAAYNTHMASSYFNAYDIRNISKVVQWNVEEVFQHSAEVIVALDDFVYASHYNDSF<br>TCKYRVDRYHILAYLTPDHNLDKRISEVEIPNQITPSPYWITPVPMTVHQQPMFNLQQNLL<br>PYQLPTHFSSPFTYAFGRMKRQIGTSPLPWLIKKLSFFKSGPRVGDLTPTGLCRRRRRS<br>PRCAIWRELVCVYPIHLSIFHDIGYAIPIFCPSTLTGLFGSIICDGGVAYDMIRIVEETRQQP<br>GFDKHNSRAMAKMITRTMERRFDTTFDSIAAEADFAWKTQKFNGRVSKLYLGGYSALSF<br>QSSPDPPPASDFVDVANDPTVDGPVYTDREQEDDDNDDDDGGDDMSSTSTTTTTTIPP<br>PFFALPVPPPPPVPPPPPPPPFLTCFSRDTWVTTPSGKKRMDEIEIGDYVLTADLKTALFS<br>AITLWIHREPETVQEFLEIKTDNGKTLQLTAGHFIYATECRYLPSKNSSLLNSTPERYRHLI<br>DTLPDDSETKLASQLKIGECLLIHNGDQFRMQKIDSISKTVSTGIYSPLTENGRILVNDVLA<br>SCYSEVQQNVLQTTFFWAFDRLRNLIVQYFGDLYLDEIELPTGTSLYKEVLTLVIPIRK
Ce	grd-12	F02D8.2	ground only	V	ADC-WF AGG-CQ	MLHRPKLTVILSVLLTFRLADCWFLSMLGGGAGGCQNQCPPAYSGYYQSRNYNPPQQR<br>FNYGLPPPTPPANSYATAPANYAAPSNAYPFAPQYSIPMNSYAMPKYAVAPQYAMVPY<br>PTPPAYVRPPPVYVTPPPVYITPPPTTTTTIPPPKCFQNTQGYKCCNRQLDQFLEQKVGE<br>MLKPEWQRCNLQRFATQLQHETQQMFNHSMEAIVASGEVQNLSNYRGDLYCKKRSRD<br>GKIVVIYGSAVPYSLDTGVTRPMNDDELRTQMYPAKYDEIGVHDGHEENIWF
Cb	grd-12	CBP17234, CBG11599	ground only		ADG-WF	MSTLLLFLLLTCRLADGWFLGMMGGGCQNRCPTYSGYYQNRSPNPYAQQPAQYALPQ<br>TPPPNQYATAPANYGQSPQAYPVAPQHAIPMNSYAGGQYAVAPSYPTPPQYPVAYPTP<br>PAYVRPPPPTYVRPPPVYVTIPPTTTTTTTLPPPKCFLNNQGFKCCNRLLDQYLDQVVTN<br>MQRPSWQRCNLQRFATQLQDEAQGLFNHSMETVVASGHMENRAQYRGDLYCKKRSM<br>DGKLVVLYGSAVPYALENGVTRPMNEDELRMANYPAKYDEIGTYDGTENIWT
Ce	grd-13	W05E7.3	ground only	IV	VLA-AY	MSRFLLLTVLVASVLAAYDDLPKPADPYQPPGTEPKTESTCPDPYKKIITQLRAELGKDV<br>SSIKFTNQLGSRVQKAFGSAHEITMGPSEATLKTNFNGTICRHASTDGFHYIVYPTPGQY<br>NINNAAVEEYFEKFAEFAALGKSANIADLPKDPRNV
Ce	grd-14	T01B10.2	ground only	X	VFA-AY	MVKLLLFVAISSATVFAAYDDLPKPADPYEPPGYEPEACPDKYSKIITQLRSDLGKDVSSI<br>KFTNQLGSRVQKAFGSAHEIMMGPSAPTLKTNFNGTICQHAGDDGFNYVVYPSPGKYDI<br>NNVAVEEYFEKFAEFAALGKGANIADLPKDPRAV
Cb	grd-14	CBP05429, CBG22788 &<br>CBP12267,<br>CBG22789	ground only		VFA-AY	MKLLLVLAISAATVFAAYDDLPKPADPYEPPGYEPEPDKPCPDKYVKIINDLRAELGKDVS<br>SIKFTNTLGSRVQKAFGSAHEVMMAPSAPTLKTNFNGTICRQAADDGNHYVVYPSPGQ<br>YDLNNLAQEEYFEKFAEFAALGKNANIADLPKDPRSI
Ce	grd-15	Y87G2A.15,Y87G2A.M,Y87G2A.j	ground only	I	AFS-VP	MHEVTVICLLVFLYSTCQIAFSVPIVVSELNGEKFSAAPNNTPITKVYIFGRPVYIREPFVIP<br>QRDEQIDFPKLIEDASRQRKHRAPYVDEPVLAAKPKYAEHKYPLKQCYTETSGFMCCNP<br>KLEKVMSETALKMKSSKSCNLQKMSSMLQAASEKAFGTDFEAIAGTGDFASKIHFYSDF<br>VCKMEREGRTMLVYATPSRHNYAMPYQL
Cb	grd-15	CBP07824, CBG08040	ground only		VVS-IP	MAATAFAENILVISSIFLISTVYLVVSIPVTHVDSAGEEKYSAAPNNTPISKVYIFGRPIYIRE<br>PFVIPQRNVSENIDFSKLFDQSKLRKNRASYTEEQGRQGNQPEQGRYNTNSYQNGYKQ<br>PADEYADEYPWPGKYPLRQCYTETSGYMCCNLHLEKVIHNATQKMKESKACNLQKMAT<br>MLADVTEDVFGTDFEAVAAVGDFASKIHFYSDYVCKMQRDGRTILVYATPSRHNGTGKY<br>SNDRNGNGGNGNGKNGGNYLVTPYTL
Ce	grd-16	Y69A2AL.1,<br>Y69A2A_6076.	ground only	IV	GSA-QK	MRCIQLYVFYLISQLFTTGSAQKTFHYGVTGGEQFGRHEPSTRLSRFQSTPVSLNPLEDT<br>LRVQQILKKLEDPGGLEIRRRSTSRRLHGVLTEPFGTPLVISGNSSEKKKKKPLKIKKRNG<br>RRNPKSPEVNEYELQLAKQIEEDQLKFVEDEKNPMKYVVENGLLFRQSRYSPLARVEIP<br>PVSGMSEISGISQRTSHSVGQTMQNPPRKRRRKQSYKLKNSKSSSESMFRTNVFRGNI<br>NRQIPAIPLPSPFSVAYGKPSFAIAREEDGKCYTNRFGYRCCDEALEALILKSYEKLRRKS<br>NDLEDNLSKIASTLRRDSRQVFAKNLEAIVSTSNFGTSIPSDFSCKVELGPNRFVAQVFV<br>PELGLDAKTTTRRHRIPYHELSREDLSDAADLVVSRNGIIVSKLL
Cb	grd-16		ground only		VSS-QK	MQLSGIPVIYYLIYQLFSTVSSQKTYSYGVTGREFIHEPSSRLSRYRSTSVSLNPLEDNLR<br>VQQILKKLEDPGGIEIRRKSTSRKLHGVISEPLGRPLVLSGGLSNDERAVTKKVRKAPVS<br>RRKEKSPEVNQFEMDLARQIEEDQLKFVEDEKNPMKYVVENGLLFKQSRYSPLARVEIP<br>QMVPPKFVLKKRKRKQRKVVKNKRIRTISSGTGEVGSVFRTNVFRGNINRQIPAIPFPSP<br>FSAAYGKPSFAIQREEDGTCYTNRFGYRCCDEALESLILKSYEKMRRRSDSLEENLAKIA<br>STLRRDSRQVFAKNFEAIVSSSNFGTSIPSDFSCKVELGPSRFVAQVFVPELGFDAKPTI<br>SRHRIPYHELSREDLSDSTDLVVSRNGIIVSQVL
Ce	grd-17p	Y102A5c.34,Y102A5.W	ground only	V	VRA-QS	MKLLLLWSFSIGVLTAVRAQSDTCPALKDRQLAKPGKLFCCDRIIKTVAETGMKTLDLYG<br>PNGPRTLGPIVQALSSLVQKHFQVAYEIVMAPNGFVLNTNYNGTRLCKFQTNSYTLAIYE<br>TPAKYDINSAR*KYFNKFALHDKLRLLPVSKHLRQLSRLAHTSHRGLLLPIPPPEHSLVGR<br>LFGR
Ce	hog-1	W06B11.4	hog only	X	no	TNGHACFSTDSWMTTPSGKKR<br>MTTPSGKKRMDQVGIGDLVLTGNLTATYYTPIITWMHREPENRYNFYTIMTEYGKMLAV<br>SAKHLIYRNLC<br>DENYAEYVKYLPKGRNVVYAEELKVGDCLVLLYKGKFRQQRVMRISITERKGIYAPITKN<br>GRIIVNDIVA<br>SVFSGIKHTRLQSDYYSTIAYAQSWLWIFGETVFHKATIPIGSALASDVLRLVIP
Cb	hog-1		hog only		no	MFLTETDGHACFSTDSWMTTPTGKKRMDQVAIGDLVLTGNLTATYYTPIISWMHREPDN<br>RYNFHTIMTEYGKMLAVSAKHLIYRNLCDENYAEYVRYLPKGRNVVFAEELKVGDCLVLL<br>YKGKYRQQRVMRISITERKGIFAPITENGRIIVNDIVASVYSGIKHTRLQGQYYSTVAYIQS<br>WLRLFGDSVFHTTAIPVGSSLASDLLRLVVP

Ce	qua-1	T05C12.10, M110	qua hog	II	VES-LN	MRRLSAILPILLLSNFWPTVESLNYKCHNDQILVVQSFGNDTIRMHCQRLDLCGYQKLKC<br>DYDELQPQCGGKLNFVSHVNQKGSTAPVEHTCCNLFNPRSHHSIPTHIGNDCFIYELPD<br>GSSNGKKVDPAPADDAPYAVLKNPAEIPEQFDGVTGYRLRLFLLKNKSPPTLLVKGIERR<br>LDGYRVTICRPRCTSYDKVVNDNEGAEDGEWKAISWSSWSSSSWSTWARHAFNKAAA<br>EGGEAAERIRTRMPIGEKTVAGAAGATGAAGSDKSNINIHVESNGNNNNSFEGGRSSSE<br>KSDGQLNREISGSSEAGAGGKGGAGADGAAGSGAGAGAGAGTNGNINITVHTDGKSG<br>GNAVAVANANVTVNGAGGVSTTGTGAQTGNESGLGGSAGTDKAGGKKGGHGDSGDS<br>GNNKNKDNGKGKGKGKNDEEDEEDNGDEDGNGKGGNGGNPKGEWDDGDGDEDDD<br>GTDGGSKESGNNGKGKGKGSGDGDGNRNGNGDGNGRPKGDGNIKINIHSPDDNDLLE<br>KDENGPNGKGGAGNGNGDGDKDNNGKGNGTGDGDGDGNGNGNGLTGDGNGTGDG<br>DNNESGNGNGDGSDKNSGAGAGTKPENREGGDGNGNGTGDGNGDGNDNGNGSKGL<br>GTGSGDGKGEGNKSGTPGKSDGKEDGAGSNGSGNGKEGDGNKSGGSGKGGAGNGK<br>SGDGSGDGKNNGNGGTGDGKDKNGKGSGSGDNDKSGTRAAGKGNAEGNGKGNGN<br>DGKGSGSGDGSGAGGKGDKSDSESGNEADGKDGKKNEGAGGEAAAGSGGANKGGS<br>DGDDDDVDVTDVEVGTKPLTGTKLEELLAKLPNETADGNATGDGNEFGTVQTGAKHNA<br>ESSASGIPLVQARSNTVNGGAPVPPAPGSGATGSGTSGSGTSESVTNGSGATESGSTG<br>SGTTGTGTSGTGSSGTGASAARTSSIAGDAPQAAVLADTPGAAGAAGGGRSNCFSADS<br>LVTTVTGQKRMDELQIGDYVLVPSSGNVLKYEKVEMFYHREPKTRTNFVVLYTKSGRKL<br>SLTGRHLLPVAECSQVEQYTMNPDGIDVAMRESKYAEKARKGECVLSIDESGEVIADEIV
Cb	qua-1	CBP00142	qua hog		VDG-LN	MRRLCAILPILLLSNFWPTVDGLNYKCHNDQVLVVQSFGNDTIRMHCQRLDLCGYQKLK<br>CDYDELQPQCGGKLNFVAHVNQKGSTAPVEHTCCNLFNPRSHGSIPTHIGNDCFIYELP<br>DGSSNGKKVDPAPADDAPYAVLKNPAEIPEQFDGVTGYRLRLFLLKNKSPPTLLVKGIER<br>RLEGYRVTICRPRCTSYDKVVNDNEGTPESEWKAISWSSWSSSSWSTWARHAFNKAA<br>AESGGSADRIRTRMPIGEKATAAGVPTGAAGSDKNNINIHVESNGNNNNSFGGNGSGE<br>KNGSGENGLNREGSGANGGAGADGAGAGRSGAGAGADGAAGAGAGTHGNINITVHT<br>DGKSGGNAVAVANANVTVNGANGKVDTTGTGASGNGAAGGNGGNGGHGDSGDSRD<br>AKKDKDHGKGKGKGDGDGDSDGGAGNGSGNGSGKDKGDGNGDSDGNGNGSGGSG<br>GSGDKKPKPAGEWDDGDGDDDESGKGTGSNEAGNGKGGDGDGDGSKGAGGSGGK<br>PDGNIKINIHSPDDNDLLEKDENGPGGNGKGDGDKDGGAGGAGGSDKDGGDGNGGSD<br>KDGGAGGDGNGDKDGNGNGNGNGLTDGGDGNGGSKNGPRDNDGNGDGNKETGAG<br>GNGNGDENGNGAGAGGNGNGDGNGGSNRPNGNGAGGDGNGTEAREGGDGNGGSN<br>GGSNGSGDKGAGAGGKGKGDGSVGDKYDKGSGGAGGDKDGKGAGGSGADKDGNG<br>GNGKSNKGDKDGNGKGSDKDKNGKDGAGKAGNGDKDGKGGKKDDSGKDGAGSGKD<br>KDAGKGKGAGDKDKKGGAGAGGKGAGGAGAGGKGAGAGAAAAGKGTGGGDDDDDV<br>DVTDVEVGTKPLTGNTLKELLAKLPNETADKDLGNGDELTNASLNRNQHAEGSTGTAPG<br>TSAAAASAPDTTGGTGGTGTTANAANTGGGTTGSGATSSGTTESGSTGSTGGSGSTG<br>GSGSTGSGSTGTGASTAQRSAVAADTPAAAAADAVAADAAGGGGAGGGGRSNCFSA<br>DSLVTTVTGQKRMDELQIGDYVLVPSAGNVLKYERVEMFYHREPKTRTNFVVIFTKSGK<br>KLSLTGRHLLPVAECSQVEKYTMNPDGIDAAMRESKYAEKAKKGECVLSIDASGDVIADE
Ce	grl-1	C24G6.7	grl, ground-like	V	AAA-NN	MLAPILLLLYFSVNSAAANNCGGYMCGSANYYQAYAQQQPATATRYQASYIPQQMPQH<br>TSQPMIQYYYPANTYQIAPQQPTLQKVSPPVISSSVDKESLVTTGKMNYDDFEKELEKLK<br>PASAVTTDEKVDSKLDLNVLNASIPTQSITYIREFPSGYSPEAFITRPLPYRPVPYMPVQL<br>PAACQQYFLPPQRPQPMPQVTPQIIQLPSAYVTAPPVTIRQQPLSILPPPINDCCGKCGA<br>PCKFRSKKNVIALASKIFTAQFVPRRDGEDEEEPKDPKCSSEKLKDLMNKYITRTVALSK<br>RLIQKNAESELGGYFSVFCSIDDFSYVARSEMFCQLQKNDITCYAFKHK
Cb	grl-1	CBP08308, CBG09376, CbC24G6.7	grl ground-like	-	SDA-NC	MLAPILLLLYFSVNPSDANCGGYLCGSASYYQQQPQQIPRYQPSYTPVQHPSQQQVLQ<br>YYYPANTYQIAPQPMPQKVTPPVISPSLDKETPVSTGKMNYDEFEKELEKLKPASAVTSE<br>DKVDNKLDLALLNASIPTQSITYIREFPAGYSPEAFMTRPLPYRPVPFVPVQLPAACQQYF<br>MPRPMPMQPQVVQLPSAYVTAPPISVRQQPVSILPPPINDCCGKCGAPCKFRSKKNVIA<br>LASKIFTAEYVPRRDADNEDEPKDPKCSSEKLKDLMNKYITRTVALSKRLIQKNAESELG<br>GYFSVFCSIDDFSYVARSEMFCQLQKNDITCYAFKHK
Ce	grl-2	T16G1.8, T16G1.H	grl ground-like	V	SFQ-FM	MLTALILLQLLIPPSFQFMFGGSGGGGCGCPCPVPPPVPICAPQTICAQQAPCSSSSSYS<br>PSYSASYASVPAPVPSPFYQSGWSNPIPSYASAPALPSYSTAYSMAPPPLILPSSPSYVA<br>PVSAPSYSLTPSISIPGPFPPAPLYVPPAPPMPIVTDGYDKISIVTSIATTPSYLQSGYAPA<br>TLAYEKQYDEESSLEGMAPPPPPPPPIDIPKDVKTYDYRTSEVVSHSENYKPAFVPSASY<br>LEDVSEEGQLVEKQGHWSKGSDSQTSSKYGQFESRHNILKRMKTESIPRTNNTCNSIKL<br>ANVMMRAIVDDVSVSKRMIQHATKSAFDGAKFDVFCAIGEFSYSIHSRKYCEVTKQEVT<br>CFAFR
Cb	grl-2	CBP02375, CBG09498, CbT16G1.8	grl ground-like	-	SLQ-FF	MLTALILLQLLIPPSLQFFFGSSGGGCGCPCPVPPPIPICAPQPICAQPAPCSSSPSYPSY<br>SPSYSSYASAPAPVPSPFYQTGWSPAIQPYASAPTIPSYSTSYSAAPPPLVIPSGPSYVA<br>PVSAPSYSLTPSISIPGPLPPSPIYMPALAMPIVTNGYDQISIVTSIATTPSYLQSGYVPAAS<br>KGYETKYDEENSVEGMAPPPPPPPIGIPKEPSTYDYRTSEVVTPSDYYKPAIVPSMSYM<br>EDSSEEGQLVEKQGYWMKGHPVSQTSSKYGQFEARHNILKRMKTEAIPRTNNTCNSIKL<br>ANVMMRAIVDDVSVSKRMIQHATKLAFDGAKFDVFCAIGEFSYSVHSRKYCEVTKQDVT<br>CFAFR
Ce	grl-3	K03B8.7	grl ground-like	V	VLT-AP	MTVKYFVILLLLIVFIIPSVLTAPVRTELCCCGCIEEPCPLVGPSCPAPRAPCNASVETVKC<br>SALQRLFEISQHKLFADEDDEETSTFQPTPSFDHQKTMKLLPTLNAKEVQRMKNEIMEIS<br>TNKTRKSEIRFSPPIESDRVNGLHRISHMMKEMEGMLMLMRDEISIQIENSSPVKLLEEAA<br>TRTEIDSSLHASPMTRAIRHKRAETENCNDEKLRKLIKENIRRDAKSSKREIQKAANKEFG<br>GHFNVICSPCEFSFVVASQKYCDGFKDDVACFAFLQPPTKLKLDDE
Cb	grl-3	CBP20597, CBG23193	grl ground-like		ILS-AP	MKNDRQNFYILLFLACFAHSILSAPVRTELCCCGCTEEPCPLVGPSCPAPDAPCNKSRET<br>VKCSALQRLFEISQHKILADVGDEEDVTTVRPTLNFEVEHQKTLQLLPTLDAREVQRMKN<br>EILEITTNRTRKAGIRLSPRIESEKVNGLHRISHLMKEMEGILSLMRDEVTTQIENSSPVKL<br>LEEAATRTEVDSSLHATPMTRGMRVKRAETDNCNDEKLRKLIKDNIKKDAKSSKREIQKA<br>ANKEFGGHFNVICSPCEFSFVVASQKYCDGFKDDVACFAFLQPPTKLKLDDE
Ce	grl-4	F42C5.7	grl ground-like	IV	ASS-TF or FG-GC	MLLPISILFSIIPIASSTFGGCCSMGPPPCPPPPPPPMCAPPPLPCPPPPICPPQFCPPPPM<br>CPPPPPPPPPPMCPPPPPPPMPSYSPCQSYAPAPVFNQYAMQPANDCCCRCGSPCRF<br>MARHRTHGSKLFTTEDPEEDPTCNSKKLRRVMERNMNGDPSISKRAIQKAVEEKMFGK<br>FNVICARGDFSYVAYTETYCQVANDDVTCYAFRPM
Cb	grl-4	CBP21652, CBG05832	grl ground-like		ASS-TF or TFG-GC	MLLPISILLSLVPIASSTFGGCCSMGPPPCPPPPPPPMCAPPPLPCPPPPICPPQFCPPPPP<br>CPPPPPPPPPPPMCPPPPPPPMYSPCQSYAPAPVFNQYAMQPANDCCCRCGSPCRFMA<br>RHRTHGSKLFTTEEQEEDPTCNSKKLRRVMENNMNRDPTISKRAIQKAAEEKMFGKFN<br>VICAKGDFSYVAYTETYCQVANDDVTCYAFRPM
Ce	grl-5	Y47D7A.5,Y47D7A.x, Y47D7A_146.b	grl ground-like	V	AQA-FF	MRTAVLLIVGFATAQAFFFGGGSSCGCSAPPACPPPPPPSPCGGGGYARAYSAPTFTA<br>GGAGYQQQPQYQPQQSSFGSSYAAPAPFAAQPAQFAVQSAPPQPIVAPPPQAYAAPH<br>KRHVDVMDIDTGVDQSEGYVARVKREENVFDPKCNSEDLKAIIVANIHESTAVAKRQIQT<br>AAADAIGGRVDVICSKGTFSYIVNTELYCETEKDGTTCFAFKQSS
Cb	grl-5	CBP12513, CBG23612	grl ground-like		SQA-FF	MLRTTLLLAIGVVASQAFFFGGGGGAACGCSAPPACPPPPSPSPCGGGGGGYARAYSA<br>PTFAAGGGSYPQQPLYQPQPSYQPAAAPAAFGSSYAAQPQPFAAAPAPFASQPQSFA<br>AQPQSFAAAPQPIVSPPQGYAAPHKRHIINNMEDTGVDQSEGYVARVKRDEEAVFDPK<br>CNSEDLKAIIIANINESTAVAKRQIQSAAADAIGGRVDVICSKGTFSYIVNTELYCETEKDG<br>TTCFAFKQSS
Ce	grl-6	K10C2.5	grl ground-like	X	AFA-QY	MSTLSVAVFLISFHFAFAQYPFGRGGCGGCPTPMCQPRMPCAAPMPMPMPMPVCPPP<br>PPCPAQFCPPPPICPPPPPPPMPCPPPPPPMPRPSCPCMMQRPSFYPSYVPQYYQPM<br>MPQPMPTAGGCGGGAVVPSVRIPAQNDCCCGCSSPCKYKSVRRAAFAAKTIDPSCNS<br>SELKNIILDNISEDASESKRNIQKIAEETLGHEVNVICGTGEFSYIAHTDTFCQAFKEDVTC<br>YAFKPLQ

Cb	grl-6	CBP01297, CBG05066	grl ground-like		VYA-QY	MSTSSLAIFLFALSCVYAQYPFGRGGCGGCPTPLCQPRMPCAAPMPMPMPMPVCPPPPPCPAQFCPPPPICPPPPPAPMPCPPPPPPMPRPSCPCMQQRPFYPSYVPQYYPQPMYQQPMPMPMGGGCSGGAVVPAVRIPAQNDCCCGCSSPCKYKSVRRAAFAAKTVDPSCNSSELKNIILDNIIEDASESKRTIQKIAEEQLGHEVNVICGTGEFSYIAHTDTFCQAFKENVTCYAFKPL
Ce	grl-7	T02E9.2	grl ground-like	V	TSA-FF	MLFRLVSTLLLAQTTSAFFFGGGGGGCGCQQAPACAPHPAPAPCSGGNIVQGYVGAPQQGGYAAAPQYPQQGGYGGAQQGFQAAPAPYQPQGGYQAGPAPVQGYQPQAQVPAGGYQAPVQPAPGPAEVAPAQVQAGGNYQDAPAQVAEVATAQESAPPPSEAAYTGEQEVVASLAREEPNYQNTGTNVIEAAQHASELGNQAAAAAAKVAEVVEEEEEPIKKKDEGKTTHMKTVKAPAAASSTTAAAASTSTEFPVGEEIVADTNEKEVDISELHLTDDPLCNSDDLRKVVIDNIDDQLNSSKRMIQLAAEAQFGGRFDVICANGDFSYVTNTELYCQETKGDISCYTYRQL
Cb	grl-7	CBP05493, CBG23176	grl ground-like		TSA-FF	MLFRLVSTLLLAQTTSAFFFGGGGGGCGCQQAPACAPPPAPAPCSGGNIVQGYVGAPQQGGYAAAPQYQQGGYAGAPQQFQQGPGPAPYQPQGGYQAGPAPVQAPAGGYQEPVPQAPQAPQPQPAEVAPAQVQAGGNYQETPAQTAGVDPAQQQSAVPENEAAYTGEEQEVVASLAREEPNYQNTGTNVVEADQHASELGNQAAAAAAKVAEVVEEDDEPIKKKDEGKTTKVKTVKTPAASPASSTTTAASSSTSTEFPVGEEIVPDTNEKEVDISELHLTDDPLCNSDDLRKVVIDNIDDQLNSSKRMIQLAAEAQFGGRFDVICANGDFSYVTNTELYCQETKGDISCYTYRQL
Ce	grl-8	ZC487.5	grl ground-like	V	ASS-AI	MCHYYLLRGFLLISVASSAIIPLPSPPNPLPEPPTVDASPSLTSEKRTVAMEVTSSAHHLLRPKTESDSEEEQPKPTRNRVRLHSHNSKVSKVSRTREVEVLSEKCNDDKLEQIMQDAMTPSLSTSKMVISERATRDFGANFDVICARGHFSYYVEAASYCEVTMNDVTCLAYKPGPQTDDGSKNDFIDIKDELIKNKNTREKDEAESKKNR
Cb	grl-8	CBP11197, CBG19006	grl ground-like		VLS-AI	MAAEFLFLLSTIPVVLSAIVPLPNPPNPIPEPPTVDTFPSLTSEKQTPIALDITSSADQQENERKVSRNRVRLHSHNSKISKLSRTREVEVLSEKCNDDELQQIMQDAMTPSLSTSKMVISERAARDFGANFDVICARGHFSYYVEAESYCEVTLNDITCLAFKPTSENSESKQETTDFANIREQLNGKLREKGEDEAKLNR
Ce	grl-9	ZC487.4	grl ground-like	V	TQA-SP	MTRSWLLVAALLAFTQASPPMCHCPNGQVGMQCPSPINFNCPPQAPCPPQQGCNMFGGLPTLPTLAPQQFQTLAPFTLAPLPGSPPGMAGPPLLPPAVAPPTNGQETLVGINNPVQPPPPVQYQNQGPQYIEAPPPPPSPPPPPPPPPPPQLQQQQVQLPLQDATTLRPSTLPSETYVELENPEAVVNEPQPIQAQTEEPATTQKATDAPPPPILQNKIERVFESEPNSGYRKPVRRQAPPLSEKCNDDRLRKIIEQNVDDNPSTSKRKIQKAAAEEIGGLFDVICSAHDFSYLANTQLFCESGNDDVTCFAFLHSLIQ
Cb	grl-9	CBP11196, CBG19005	grl ground-like		VNA-SP	MTISWLLAVVLLSSVNASPPMCHCPNGQVGMQCPAPINFNCPPQAPCPASPPCNMFGGLQPLPTMAPQQFPTFAPFTLSPLPGSPPGASGPALLPPAVSPPTNGQETLVGINNQNQPPPPPVQYNQGPQFVEAPPPPPPPPPPPPPPPPPPLQQQIPVQDATTLRPSTLPSETYVELENPEAIINENAAQVQKNPEEARNNVEKVTDEPPPPVLQNKVERVFESEPNNGYRQPVRRQAPPLNDKCNDDRLRRIIESNVDDNPSTSKRKIQKAATEEIGGLFDVICSAHDFSYLANTQLFCESGNDDVTCFAFLHSLIQ
Ce	grl-10	C26F1.5	grl ground-like	V	VNG-FF	MRHSLTLVVVILSLSLYSVNGFFFGAAGGGGACGCAPRPACPPPPPSGCSGSGVRAVARGAKTMTFDQPIYSPTPQSYAQPPRVYSVPDELDLRAAAFGVPIQPQPQYTIFDILHNLGESNDNPAQQLVVADGSTNTNYFPKENDEDLTVSDMTGDGGLYRSKNIRRAPVEPTVHVHRDSSVETTTISSEISGMETDETLDKNKCSSSVLRKLMIENISDSSSESKRNINLAAEGKFGGNVDVICSRGHFSYIFTSNLYCEATRGLTTCIAFRQSDKSRRRR
Cb	grl-10	CBP04840, CBG20585	grl ground-like		VNG-FF	MRHSVTLFILLTSSSVNGFFFGAAGGGGACGCSQPPACPPPPPPCGAHRAVARGAKTRSFDQPITAGYQPNYATNPGPQVYTVPDELDLRAAAFGVPIQPQPQYSIFDVLHNLGESRDSPAQQLVVADGSTNTDYFPKENDEDLTVSEMTGSGGWYRAKEIRRAPAEPTVHVHRDGSVESTTLGSVATEESFDKNKCSSAVLRKLMLEEITDSSAESKRNINVAAEGKFGGNVDVICSRGHFSYIFTSNLYCEVSKGLTTCIAFRQSDKVRRRK
Ce	grl-11	ZK512.9, ZK512.x	grl ground-like	III	ISG-IQ	MYFSIFSVFIGLQITVEISGIQLIDGSTNVTVFHNDYTDSYSTSLIQNPNIRTKPIPRSRKDDDCSDELLRKVMNEHISDEGMTESKRAIHEAARRDFEGTWSIICAPCAFSYLAHAQDYCIHSRHGITCLLYRDG
Cb	grl-11	CBP07471, CBG06938	grl ground-like		VRG-QK	MALSCYRILLVLIGLRVTIEVRGQKTMIDGNTNLTSSRQYDYIEPYPGSLIQNPNVRTKPVVRPRKDEDCSDEILRKVMNEHISDEGMTESKRAIHAAARRDFEGTWSVICAPCAFSYLAHAQEYCIHSRHGITCLLYRDG
Ce	grl-12	F28A12.2	grl ground-like	V	ASA-FF	MLSPFLLLFLLFSAPASAFFNSLFGGSNCGCSCTPPPSTCPTTCLPIMTCTPPSPPACCNTCGTCNGKRRRRHLTMLSNATYVADHEKVNRVKRQDDQETTVTSGNCNSVELQRIIESKIDRVTAIAKRRIQEEAEATMGGRFNVICARGDFSYVANTELFCQHSVGDVTCFLFKQLSDVVRRRLM
Cb	grl-12	CBP00353, CBG01466	grl ground-like		ISS-FF	MLTLPFFLFLLLQPISSFFFPSGGGSNCGCTCTPPPSTCPTVTPCAPIMTCSPPPVQACCNTCGTCKGRRKRRHLMLSNATYVASHDPARRVKREDDTEAASDGRCNSEELRKIIENKIDRVTAIAKRRIQEEAESTMGGRFNVICARGDFSYVANTELYCQHSVGDVTCFLFKQLSDVVRRRLM
Ce	grl-13	F32D1.4	grl ground-like	V	ANS-LF	MVCVLVLILMASLTANSLFFNGGGGCGCRPQQCGCAAASLPVRSYCPPSPCGRQFQSFPSYTQPYSPVSFRPPPPTIIQQQPYYSVPHPIPIQTQQFHNSYQQTPVFTSSYNAPPFASSSSYSNAPNSFQNAPIQVASAKETSADTFDTLIQKSKPETTDMKNDLDVDVNALSNFETSLQAYKSMNRNGIRRAQAAKTEKAGKCSSERLQQIMEEAMSSNVSVSKLKISRGAKKEFGYNFDVICSQFDFSYLISSNIFCRVELDGQICLAYEN
Cb	grl-13	CBP12521, CBG23622	grl ground-like		SQG-LF	MVHILLLSMTTIGISQGLFFGGGGGCGCRPQTCGCAAPLPVARSYCPPTPCGPRPFPVYPSYQPVSFRPPPPTLIQQPYYAQPQQIPVPIQAQFHNSYQQTPVFTSSYNAPFNNNQLPSYINSPTQNAPIQVTSAKETTADSFDTVIQKSRTDTSPAKNDLDVDNALSNFETSLQAYKSMNGIRGIRRAQGAKIEKPEKCSSTRLQQIMEESMSSSVSVSKLKISQGAKKEFGYNFDVVCSQFDFSYLISSNIFCRVELDGQICLAYEN
Ce	grl-14	T03D8.4	grl ground-like	V	ISA-KS	MILILITLVFVFQKTQISAKSDFEILKNDSIFDGGTQGNKAFLKTQLLRNLNNFYPSINNGEKLEMLTGRKPEFEDDRERNQNFDGNSVTEPFPTQYPTLIPYARESNPEEELQLATAPTSKIRSEGRITSEQKMDSIRDFRMKLYKAFKNRPKLSRMIRKSNVNDVVEMNDGFPTIMDKNRQIILSRTEPNWQSLNTRKPNQTYGRDQNGNLIPLLGPYPPREIRYAYRTTNRQPVVYVPSASPVNQPSHASNTVPVAVASYLVTANPSSQPIQLHILPPIPSPQPQIIYQQTTPSSQFVAYSTFAPPIEPSNECGNGQCKPESDEDKCNSQRLRDIIFNNIVSGDAEASKRAVQSAAEAETGLFFDAICGTGFFSYIAHTDEFCLASSGGVNCYVFAPICQIDSQNQQKRTSKKLVKLSKN
Cb	grl-14	CBP01428, CBG05569	grl ground-like		VDS-KQ	MKVTRRVTVDGVSTVTLLLALYTLVDSKQSDFGILKNDSIFDGGTQGNKAFLKTRLLENLNNFFPSINNEERLEMLTGSSKSKEEDLDDSNQELTDNSVTAPPPPEFPTLIPSSEPEEEFPTSSVESSKIRSEGRITSETKLDSIRDWRKRLYKAFKNRSKMSRIIRKSPSEEVVEMNDASPTIMDKNRQIILSRTEPNWQSLSSGKHIQTYGRDPNGKLIPLPYPPREIRYAYRTTHRQPVVYVPAAPAPAPPQNTIPVPVASYLVTANPSPQPIQLHILPPIPSPPAPQPQIIYQQTTQAPQFFTFSTFAPTTDNQNDCSNGQCRPESDDEKCNSQRLRTIIFNNIVNGDAEASKRAVQSAAEADTGLFFDAICGTGFFSYIAHTDEFCLASSGGVNCYVFAPICQGGDEQRKKKLSKN
Ce	grl-15	Y75B8A.20, Y75B8A.W	grl ground-like	III	VSC-TF	MQSFLQIFLIFSVISTVSCTFFNGGCGCQPPPICLPPPPPMCFTEIQLPPIRIPIIPLPRIELPQPCCPTCACGGRKKREIDAGVQEEESVSTKDVSCNDDSLLAIMKKEMTTGESSAVKIALVEAAEQELGGRFTVVCSQGAFSFVTSTTSYCLHSQAGLNCYLFKTQ
Cb	grl-15	CBP12825, CBG24285	grl ground-like		VSS-TF	MQNLARILTISAVFGSVSSTFFNGGCGCPPPPLCLPPPPPMCFTEIQLPPIRVPVIPLPRIELPQPCCPTCACGRKKREIDTGVAEDTVSTKDVSCNDDGLLAIMKKEMTTGESSSVKIALVEAAEQELGGRFTVVCSQGAFSFVTSTTSYCLHSQSGLNCYLFRTQ
		Y65B4BR.6,	grl ground-			MRVLVAVLLAVSPALACIGGAGSAGGCCPPSQPSCGGGAPPCSSSSYATGGGGGSYAAAPALPPPPPPPPAPIGGGAGYAAPFGGPGPVGGASYSQGPQGFGGQVGGGAYQAGPGPIGGGASSHAGSQAGPSYAGASQAVGASSYARSQGPIGGGPSYAGASQPAPVAPQGGYNAGPSAPVEQVQQGGYQSGPVSSIQVTQVSQGGNYQAPPEHQAVQVVEQVAPVATYTQGPVPTPVEQVVETVVEPQQAPEPEQAPEPQQAPEPTVVETVQEEIVPEIVAETEAPVVVAETEAPAVVETEAPVVIEETEAPVVVEETPAPVVEETQAPVAAAKEESTYDDIVEEQPSTATETATEQVDTVEETNEAVEYADEEGDEGNCDDPELRAIVEAALKNEKDNLEAA

Ce	grl-16	[illegible] Y65B4B_137.B	[illegible] like	I	ALA-CI	[illegible] RKIEGDASAKFGGRFNAIVSDAEFAYVNWYGKRNCQLRYENRHSLTWED
Cb	grl-16	CBP01307, CBG05117	grl ground-like		ALA-CI	MRVVLAVLLAVSPALACIGGAGSAGGCCPPSQPSCAPATPPCSSSSYASGGGAYAAAPAALPPPPPPPPPSAGYAGAAGPIAGGAYSQGPQGGFGGAPQGGYQSGPIGGGQQGYAGPGPIGGGAPQGPSYAAGPAQGPIGGGQQQGPSYAAGPAQGIQGGGQEGYAGQGAAQGGQGYQAGPAQVAPVEQPAPQQGGYQSGPVNSIQVTQVSHGGLYQAPPEQPAAPQETVTEAATVVVEETQAPIIEETQAPVVEETQAPVVEETPAPAPVEQAPVAAAKEESTYDDIVEEQPATATEAATEQVDTVEETNEAVEYADEEGDEGNCDDPELRAIVENALKNEKDNLEAARKIEGDASAKFGGRFNAIVSDAEFAYVNWYGKRNCQLRYENRHSLTWED
Ce	grl-17	C56A3.1	grl ground-like	V	CHA-IF	MMLRLLLLSVSTLTLCHAIFFGMGGGGGGGGGGCCCGCGTPQPASCGCAPACPQAPSCPVCPPPQPCPAPPAAYCPQVQPVYVQSGGGGCGGGGCGGGGGGCGGGGGCGGGGGGGCGGGGGGGCGGGGGGGGGGYASGGSGGFASAPVSLPAPSYGGPPPPAPSFSHAPSGGYSSGGSSGGGYSSGGSSGGGGYAGGAAAAGATAAQVDEASETSAGEPPVDQVFHSPKEPCTQQVKYIMLRSRKVPGGGATELVEEELEQVENPPPPVEATANPLDAQLGEDIAAQQQAATEDDGDDESASSGDFKARAAKTAVTDEKCNSKILQKLVLTNIAANDALASKKAIHDNALQQFPDSSVDVICSTTGFTYLVSTTEHCEAQKDGVICFVYKRPL
Cb	grl-17	CBP02782, CBG11540	grl ground-like		CHA-IF	MMLRLLILSVSGLTLCHAIFFGMGGGGGGGCCCGCGTPQPPSCGCAPACPQAPACPVCPPPQPCPAPPAAYCPQVQPVYVGGGGGGCGGGGGGCGGGGGGGCGGGGGGENCSEGGCVGGSGGGGYASGGSGSSGGGYGRAPVSLPAPSAGYSGPPPPIPSSGSGYSSRYSSGGSSGGSGYSSGGSSGGGYGAAAAGATAAQVDEATESDGEPPADQVFHSPKEPCTQTVKYIMLRSRKVPGTETTELVEEELEQVANPTHVEATANPLDTQLGEEIAAQEQAATGNSEDTEDAESASSGEFKARAAKTAVTDEKCNSKILQKLVLSNIATNDALASKKAIHENALQQFPDSSVDVICSTTGFTYLVSTTEHCEAQKDGVICFVYKRPL
Ce	grl-18	T05C3.4	grl ground-like	V	TTA-IF	MAKYLFFYLSLVLYFHETTAIFFPQLGFGSMNCQCQNSCSSPPAQLGCLCPPPMPCYQQPTQNYYTQPPSVIYPSYQNQQPLQPSSSYLANIPPFMQSYPQPPLFTTPPPLPQDNSILIWPIDQKIRKLIYKINYPTPSQTFIPQQTLKTSYSTSFGSLGPPAPSAEKLNLQVPPALKQKEETSDQQVNEKNYVFGAHQPLSDIFYETGASEADNEIDQNYSPIITTTEAVYRPIETTAKRNYETSTHRTSSSSRATKQESRRYETELMRGEGNSDISSSGYGFESASTTSAPILLEFLEQPTKYENRSPNNTELADVTAFTQEVDEFYKSSTSSPAQTFEYGAVRDARDSTGKDETSTPKFPFDRDENVNKWTSKRKIREKEEINSKCNNPILKDLMEMKMTTSPSISKQMIYSAATEMWMGRNVNVICSKHSFSYVVVTSPIFCEHRKKALTCFVFFQP
Cb	grl-18	CBP16517, CBG09384	grl ground-like		SGA-IF	MAKNLFFFLSLVFYLHESGAIFFPQLGFGSMNCQCQSSCNSPPSQLGCLCPPPMPCYQQPAQTFYTPPPPPVIYPSYQNQQQPQPSSSYLANIPPFMQQYAQPSFFSTPPPIQVQQLPPGTSSLNYPSASQMFIAEQSLKPSYSFPGSAGPPASSDSQETLNLQPPAIKQKEETFERPVSEKNYVNTADRPLSDIFYETGASEDDKEIERNHLPILTSTEAVYRNTDTATRTNSESSTYPSSTIAVTTKRYETALKRGEGNSDISQSGYGFEAASTSEPIMLEFLEHPRKYENQSPNNTDITDVPDFTKEVDAFYKSSTSRPIETFEYGAVRDARDSSDKETSTPKFPFDREEEVSPKSKWTSKRKTREKEDSSKCNNSILKDLMELKMTGSPSISKQMIYSAATEMWMGRNVNVICSKHSFSYVVVTSPIFCEHRKKALTCFVFFQP
Ce	grl-19	R02D3.6	grl ground-like	IV	AFA-FF	MFLKLLLFLSICHVAFAFFFPRVTPNDGCGCGCGGGGGGGGCCAPPPPPPVCGGCGCGGRKKREIGHVKGHLAHRDNDQEWNNQCNSLEFSDVIVKHLRTKSLSTSRDFIYKELDTAFPDSMFTVFCLQNSTVSYQADAKRYCMEKTADRSCYVFEF
Cb	grl-19	CBP05381, CBG22548	grl ground-like		ASS-LF	MLKLLILLSLCHVASSLFFPSVSPAGGGCGCGCGGGGGGGGCCAPPPPPPVCGGCCGGRKKREIGHVKGHIVHRDEQEWSNQCNSQEFSEVILKHIEMSSLKSSREAIYKELDAAYPDSMFTVFCLKNSTSSYQADAKRYCMEKSKDRMCYVFEF
Ce	grl-20	C23H5.9	grl ground-like	IV	TQA-FL	MRVLVVVSLIVVSTQAFLFPGGGGGGGCGGGCGGGCGGGGGGGCLPTLGCSVISFNVPTLKLPPPPPPPPCGGGCGCGGRKKRAASEDSKCTDPELRKIILNGVRTTTSESRDNIVASLKEKYAGVRYLVTCIEGDHDFASSSTDYCADGSQQQTCIVAKATDE
Cb	grl-20	CBP02581, CBG10552	grl ground-like		TQA-FL	MRFVVTLALIIVSTQAFLFPGGGGGGGCGGGCGGGCGGGGGGGCLPTLGCSVISFNVPTLKLPPPPPPPPCGGGCGCGRKKRAASEDSKCTDPELRKIILNGVRTTTTESRDNIVASLKEKYAGVRYLVTCIEGENDFASSSSDYCADGSQQQTCIVAKATD
Ce	grl-21	ZC168.5	grl ground-like	IV	SSA-FL	MVHSSVLIVLSLTASSSAFLLEGLGLGLGPQRPQQCCCPPPPPPPPPCGGGYEAPPPPPPSYAGGPSYAGPSYAGSYGPGPYRAKSKRSVDMKASSGDLFCNSVEVRDIIKKGMTSGEKESRETITALLKTEMNREYVVICTKQPFEYLASSDSEFCSVTNDSGITCSSFVF
Cb	grl-21	CBP10608, CBG17648	grl ground-like		TSA-FL	MVRNSVLVILSLTTATSAFLLEGLGLGLAPPRPQQCCCPPPPPPPPPCGGGYEAPPPPPAYAPPPPPPPQPYSTGPSYAGSYGAAGPYKRARSKRSMELKASSGDVLCNSVQVRDIIEKGMTSDEKESRETVVALLKAEMEREYVVICSKQHFDYLASSDSDFCSVTNDAGITCSSFVF
Ce	grl-22	W03A5.3	grl ground-like	III	TSG-FL	MLRLALVLSVFIVSTSGFLFPMAGGGGGGGGGCAPAAPACAPPPPPMCGCAPPPPPPPPPPMCGCGCGRKKRSVDDVEGVINMDSDVECNNEELREVLENNMKSTPSDSLVSVRSNLPTDQYFVTCTHGLTAYSAPAGTKSCAVRKESHFCQIFSLTNNSSNL
Cb	grl-22	CBP07014, CBG05173	grl ground-like		SSA-FL	MLRFTLLSIFIASSSAFLFPMAGGGGGGGXXXPPPPACGCGAPPPPPPPPMCCAPPPPPVCAPPLPPPPPPPMCGCGCGRKKRSVEGEPAVEGIINMDSILECNNQDLKGILEKNMKTPSDSLLSVRSSLNSDEYFVMCTHGLTAYSAPAGTKNCAVXKENHFCQVFSLNESH
Ce	grl-23	E02A10.2	grl ground-like	V	ATA-FL	MRKSIIILLLGLSVATAFLFPSGGGGGGGGGGCGGGCGGGGGCGGGGGCGGGCAPPPPPPACGGGCGGGGGGCGGGGGGCGGGGGCGGGGGGCGGGGGGCGGGGGCGGGCAPPPPPPACGGGCGGGGGGCGGGCGGGGGGGCGGGGGGGCGGGGGGCGGGGGGCGGGGGGGCGGGGGGGCGGGGGGGCGGGCGRKKREAINVVGHDDLNKCNNEELRIILNENTKETVAESIKTLKEKVAGQDYIVVCNEKPAPFTAETDDFCSLQKENVHCTILRINHKEVAEKNEEDKKEEEPKKEEEKKEEVEKKEEDEKKDEEPKKEEEKKEEEQKEEVEKKEEEEKKDEEPKKEEEKKEEEEKKEDEVEEKSEKVEEKELEPKKDEEETKKN
Cb	grl-23	CBP12461, CBG23530	grl ground-like		ATA-FL	MRKSIIILLLGLSVATAFLFPSAGGGGGGCGGGCGGGGGCGGGCGPPPPPPCGGGCGGGGGCGGGGGCAPPPPPPCGGGGGGCGGGCGGGGGGCGGGGGCGGGGGCAPPPPCGGGGGGGCGGGGGGCGGGGGGCGGGGGGCGGGGGGCGGGGGGCGGGCGRKKREAVNVIAHDDLNKCNNEELRIILNENTKETVAESIKALKEKVAGQDYIVVCNEKPAPFTAETDDFCQLQKENVHCTILRINHKEIVEEKKEEEKKVEEEPKKEEEKKEEEKKEEGSKEESKEEKKEEESKAEKKEEEPKKDEVEEKSEKAEEKELEPKKEEEEETKKN
Ce	grl-24	F11E6.2	grl ground-like	IV	ASC-QD	MSSLLLLLVLVMFFTLASCQDDNFEGERCNDVILYDIIKKASKKTDDPVIIRRTSMDTMQNVFPLARSMGCICTDRNFQFPDFTNHRYCSVRVSNFRCHAIVF
Cb	grl-24	CBP05755, CBG00435	grl ground-like		VSG-QE	MCTFHILPAILLFVVLVSGQEEMMDGEKCNDVILYDIIKRASKTTSDPAEIRKTSMDTMQRVFPLARSMGCICTDHEFQFPNFTNHRYCSVRVSNLKCHSIVF
Ce	grl-25	ZK643.8	grl ground-like	III	VSS-FL	MATYLRLVVLFLLTIHVSSFLFPSAGGGGGGGGGCGGGCGGGCGGGGGCAPPPAPCGGGCGGGGGGCGGGGGCAPPPAPCGGGCGGGGGGCGGGGGGCGGGGGCGGGGGGCGGGGGGCGGGGGGCGGGGGGCGGGGGGGCGGGSSGGCGGGSSGGCGGGGGGGCGGGGGGGCGGGSSGGGGYAVAPSGGGGCGGGGSSGGGGYAVAPSGGGGCGGGGSSGGGGYASAPSGGGGYATSGGGGSGGYATGGSSGGGYSSGGSSGGGYSTGGGGGYAGGGGGGGGGSSGGYAGSSGGGGYSAPAAAPPPPPPPPPPPAPAPVSSGGGYSEQSSGGGGGSSYSGGGEASSSSGGGYSGGGESSSSGGSSYSSGGDSSSSSGGGYSSGGDSSSSSSSSGGYSGGSDSSSSSSSSSGGYSSGGGDAGASSGGESSSAGGYSGSSSSGGEASSGGYSGGSSEPAPAPEAAPASSGGYSGGSEAAPEAAPAAPSGGYSGSEAAPEAAPAAPSGGYSGSEAAPEAAPAAPSGGYSGSEAAPEAAPAAPSGGYSGSEAAPEAAPAAPSGGYSGSESSAPAAPEPAPSSGGYSGGGGDAGSAAGGSNYSGGGETAPAAPPPAPEPAQTYSGAGGESAAAAPAPSGGGYSGSGGAGGAPNGANYDEAQEDVEEYEGGAAGFRNRRFSKGGTADDTPICNSVRLRKLIRQSLTEDPESSMQRLAQRVKSRLVSGEFYVACGEQGLLPLAGENREHCFIKSGKFACYVLRKA

Cb	grl-25	CBP21805, CBG06865	grl ground-like		VSS-FL	MATYLRLVVVLILTVHVSSFLFPSLGGGGGGGCGGGCGGGGGCGGGCGPPPPPPCGG<br>GCGGGGVCGGGGVCAPALPPPPPCGGGGGGCGGGCGGGGGGCGGGGGGCGGGG<br>GACGGGGGGGCGGGGGGCGGGGGGCGGGGGGCGGGGGGCGGGGGGCGGGSS<br>GGCGGGGGGGCGGGVKDTSRTFWNLLEVTHININEVMSSGGSSYGGASSSSGGTSYSS<br>GGDTASASSYSSGGESSSSSSSGGYSGASTGGESSSGGYSSQGSTGGESSSSGGYS<br>SGSSTGGESSLSGGYSSGGSESVSTGGESSSSGGYSGGSESTGSESTSSGGYSGGS<br>DSSATSSSSSSGGYSGGGDAGTSTGGESSSSGGYSGSSSSSSGGESSSAGGYSGSS<br>SSSESIPEPAPEAAAPAPEAAPAASYGGSESSAPAESAPVESAPAASSYGSSESAAPAA<br>PSGGDYSSSGSSESAAPAAPEPAPSAGGYSGGGSDAGATSGGGSNYSGGGDASAAA<br>PAPAPAQTYSGGGDAAVAVPAAQQGGYSGAGGAPNGANYDEAQEDVEEYEGGSAGF<br>RNRRFSKGGAADDTPICNSVRLRKLIRQSITEDPESSMQRLAQRVKSRLVSGEFYVACG<br>EQGLSPIEGEHREHCFIKTSTFACYVLRKA
Ce	grl-26	K02D7.6	grl ground-like	IV	HG-CAG or CAG-LF	MFSNSAKIVLIFAILFFDLCHGCAGLFGGGGGGGCCCQSACGRKKRSIDEDERREFEFR<br>GIASKNEDLLCNSPEMKAMIQAHMKSNPQASSKSLQSALEDHDSHRYVVVCSENPFHY<br>SIKHDSAYCGARNGSHYCQAFAI
Cb	grl-26	CBP12179, CBG22544	grl ground-like		CNG-CA	MSKIATLCLFLVLLDVCNGCAGLFGGGGGGGCCCQSGCGRKKRSVDDEEAPGFQFRGIA<br>SKADDMLCNSPDMKALIQAHMKSSPQASSKSLQVALEDHDQHRYVVVCSENRFHYSVK<br>HDSAYCGSRNGTHYCQAFAI
Ce	grl-27	F40C5.3	grl ground-like	V	TAA-CP	MQSSLLILLAIVSITAACPGLFGMGGGGGCGCGAPPPPPACGCGGRKKRSLPEKPEFFG<br>IAAGDQDVMCNTPELKKIILENTQTTAVDSSKAINTVLESRQLQRFVVVCSENPFVFTIRA<br>DTAYCGASKNGHNCHAFAM
Cb	grl-27	CBP05966, CBG01299	grl ground-like		CAA-CP	MQSSILILLCLVSICAACPGLFGMMGGGGGCGCSAPPPPSSCGCGGRKKRSLPEKPTFF<br>GIAAGDDDMMCNNTELKKIILENMQSTSVDSSKAVNGALEAKQLNRFVVVCSENPFVFT<br>VRADTSYCVARKNEHNCHAFAM
Ce	grl-28	T24A6.15	grl ground-like	V	TAA-CP	MHSSILLLLAFTSITAACPGLFGMMGGGGGCGCGAPPPPSCGCGGRKKRSLPEKPTFY<br>KISASGEDLVCNTPELKKIIVENMESSAIESSKSVNAVLETRQLNRFVVVCSENPFAFTAR<br>ADSAYCGAAKNGHNCHAFAM
Ce	grl-29	T24A6.18	grl ground-like	V	TTA-CP	MISSLLFLLVFSSITTACPGLFNMLGGAGGCGGGGGCGGGGGCGGGCGYGGGGGCG<br>YGGGYGCGIPPPPPPPPPQCGCGRKKRSLPEKPTFFGISASGDDVMCNTPELKKIIARNM<br>QDSAMDSSKTVNAALENKKLQRFTVICSENPFVFSIRADTAYCGVQRNGHNCHVFAM
Ce	grl-30p	T24A6.3	grl ground-like	V	TAA-CP	MHSSILLLLAFTSITAACPGLFGMMGGGGGCGCGAXPPPSSCGCGGKKKRSLPEKPTF<br>HGIAASDEDVMCNTPELKKIINENMQASAVDSSKAINGALESKELNRFTVVCSENQFVFTI<br>RADTAYCGAKNNGHTCNVFSM
Ce	grl-31	T24A6.19, T24A6.y	grl ground-like	V	TTA-CP	MHSSILLLLSFASITTACPGLLNIFGGAGGGGCGGCGGGCGGGCGCAPPPPPCCGGGC<br>GGCGRKKRSLPEKPTFVGIADSGDDVMCNTPALKKIILENIQTTASDSSKAINTVLESRQI<br>QRFVVVCSEKPFVFTFRADAAYCGATKNGHNCHAFAM
Ce	grl-32p	T24A6.x	grl ground-like	V		NIQTTAVDSSKAINTVLESRQLQRFVVVCSENPFVFTIRADTAYCGASKNGHNCHAFAM
