# Supplementary material for: Comprehensive analysis of gene expression patterns of hedgehog-related genes
Source: BMC Genomics. 2006 Oct 31;7:280. doi: 10.1186/1471-2164-7-280 (PMC1636047; doi:10.1186/1471-2164-7-280)
Supplement: Additional file 2 — List of primers used for generating the reporter constructs and all transgenic worm strains. [file 1471-2164-7-280-S2.pdf]

Additional Tabel 2. List of worm strains and primers used to generate the promoter::GFP constructs. The size of the amplified promoter region is indicated in base pairs.

Gene name	ORF	Worm Strain	Primer A* (5'-3')	Primer B (5'-3')	A*_B size (bp)
wrt-9	H02F09.1	TB1612	CGCTGCAGCGTTGCTGGGACTTCTTCT	ACGCGGATCCCGCATTCTGCAACTTCTAATG	2660
wrt-9	H02F09.1	TB1621	CGCTGCAGCGTTGCTGGGACTTCTTCT	ACGCGGATCCCGCATTCTGCAACTTCTAATG	2660
wrt-9	H02F09.1	TB1624	CGCTGCAGCGTTGCTGGGACTTCTTCT	ACGCGGATCCCGCATTCTGCAACTTCTAATG	2660
wrt-9	H02F09.1	TB1613	CGCTGCAGCGTTGCTGGGACTTCTTCT	CGGGATCCGAACCACAGTACGAGGCACC	2735
wrt-9	H02F09.1	TB1622	CGCTGCAGCGTTGCTGGGACTTCTTCT	CGGGATCCGAACCACAGTACGAGGCACC	2735
wrt-9	H02F09.1	TB1623	CGCTGCAGCGTTGCTGGGACTTCTTCT	CGGGATCCGAACCACAGTACGAGGCACC	2735
wrt-10	ZK1290.8	TB1624	GCCTGCAGACTAACATGGCGAGGCACT	GCGCGGATCCAGCATCCTAGCTATTTGGAAA	771
wrt-10	ZK1290.8	TB1625	GCCTGCAGACTAACATGGCGAGGCACT	GCGCGGATCCAGCATCCTAGCTATTTGGAAA	771
wrt-10	ZK1290.8	TB1616	GCCTGCAGACTAACATGGCGAGGCACT	GCGGATCCAGTGAGGGCACACGAGTTTA	1725
wrt-10	ZK1290.8	TB1620	GCCTGCAGACTAACATGGCGAGGCACT	GCGGATCCAGTGAGGGCACACGAGTTTA	1725
wrt-10	ZK1290.8	TB1622	GCCTGCAGACTAACATGGCGAGGCACT	GCGGATCCAGTGAGGGCACACGAGTTTA	1725
grd-1	R08B4.1	BC13005	TGGATCCTGATCCTAAGGCA	AGTCGACCTGCAGGCATGCAAGCTTAGCGGTGTTCATAAACGGAG	229
grd-2	F46B3.5	BC13023	GCAATTACTGAAAATTGCCGA	AGTCGACCTGCAGGCATGCAAGCTACTCGATCTGTGCACGTCTTT	2939
grd-2	F46B3.5	BC15192	AACAGATTTAGACGGCTTTTGAA	AGTCGACCTGCAGGCATGCAAGCTTGATAATGCCAGCGGATAAAG	2866
grd-4	T01B10.1	BC12830	TCATTACTCAACTTCGTTCCGAT	AGTCGACCTGCAGGCATGCAAGCTTGATCGCTGCTGAATACACAC	1115
grd-4	T01B10.1	BC12829	TCATTACTCAACTTCGTTCCGAT	AGTCGACCTGCAGGCATGCAAGCTTGATCGCTGCTGAATACACAC	1115
grd-5	F41E6.2	BC12951	ACTGTGTGAAGTAAACCGAAATGA	AGTCGACCTGCAGGCATGCAAGCTACGATGAGTGAACGGATTTCT	2885
grd-5	F41E6.2	BC12828	ACTGTGTGAAGTAAACCGAAATGA	AGTCGACCTGCAGGCATGCAAGCTACGATGAGTGAACGGATTTCT	2885
grd-6	T18H9.1	BC15234	AATGGCACATATCCGTCGAT	AGTCGACCTGCAGGCATGCAAGCTCGTGATCCGAATTGTGACTG	2058
grd-6	T18H9.1	BC13024	GCGATGAAAAGAGACAATTACCTT	AGTCGACCTGCAGGCATGCAAGCTCGTGATCCGAATTGTGACTG	2939
grd-7	F46H5.6	BC13025	TCTCAGTGCTTCTGAAAATTATGG	AGTCGACCTGCAGGCATGCAAGCTAAGACGGCATGATATCGACTAAA	2901
grd-7	F46H5.6	BC13025	TCTCAGTGCTTCTGAAAATTATGG	AGTCGACCTGCAGGCATGCAAGCTAAGACGGCATGATATCGACTAAA	2901
grd-8	C37C3.4	BC12824	CTTCCTATACTTTCCCCTCAGACA	AGTCGACCTGCAGGCATGCAAGCTTGATTCCTTGCACATCGGT	905

grd-8	C37C3.4	BC12823	CTTCCTATACTTTCCCCTCAGACA	AGTCGACCTGCAGGCATGCAAGCTTGATTCCTTGCACATCGGT	905
grd-8	C37C3.4	BC15238	CATGGCATGAAATACAAGACAAA	AGTCGACCTGCAGGCATGCAAGCTAAGTGGAAGTAATGGCACGAA	110
grd-9	C04E6.6	BC12961	TTCGACGCATTTTTATTTTCG	AGTCGACCTGCAGGCATGCAAGCTCTATCCGAATATCGCCACGTA	1146
grd-10	F09D12.1	BC12963	TCGTCATCTTTTTGTCGCTG	AGTCGACCTGCAGGCATGCAAGCTACGACGAGAGAGCGGATTT	799
grd-10	F09D12.1	BC14241	TCGTCATCTTTTTGTCGCTG	AGTCGACCTGCAGGCATGCAAGCTACGACGAGAGAGCGGATTT	799
grd-11	K02E2.2	BC12825	TCAGAAAAATTCAGAAATGGTTTT	AGTCGACCTGCAGGCATGCAAGCTAATAGCAGGAAAATGATGGAATAA	1951
grd-12	F02D8.2	BC12962	TTTCAAAACAATGTGTATCGACG	AGTCGACCTGCAGGCATGCAAGCTTCGGCCTGTGTAAGATTTCTG	2905
grd-13	W05E7.3	BC12966	TCTACTTATTCCATAACGGCCAA	AGTCGACCTGCAGGCATGCAAGCTAACGGCTGATGATCTCTGAAA	723
grd-13	W05E7.3	BC12831	GAAGGCTTGCTCATTTACCATC	AGTCGACCTGCAGGCATGCAAGCTGAGAAAACGGCTGATGATCTCT	879
grd-13	W05E7.3	BC15195	GAAGGCTTGCTCATTTACCATC	AGTCGACCTGCAGGCATGCAAGCTGAGAAAACGGCTGATGATCTCT	879
grd-14	T01B10.2	BC12965	TTTTTGTTCAGGTGATTGTCA	AGTCGACCTGCAGGCATGCAAGCTTGACTTCTGTAAGACGGAGGC	1859
grd-14	T01B10.2	BC14199	TTTTTGTTCAGGTGATTGTCA	AGTCGACCTGCAGGCATGCAAGCTTGACTTCTGTAAGACGGAGGC	1859
grd-15	Y87G2A.15	BC12964	AATAACAATCTCGCTATCCTCCA	AGTCGACCTGCAGGCATGCAAGCTAAGACGGGCGGTTCTTACTTA	841
grd-16	Y69A2AL.1	BC15226	TATTGCAAACCCTTCTCGCT	AGTCGACCTGCAGGCATGCAAGCTTTGAATACAACGGATTTTGGG	2866
grl-1	C24G6.7	BC15287	CGATGGCAACGATGAATAAA	AGTCGACCTGCAGGCATGCAAGCTCTTTCATTGCGTCATTCTTTT	287
grl-2	T16G1.8	BC12852	TGAACTTACCACACCTGCACA	AGTCGACCTGCAGGCATGCAAGCTAAGTGCAGTTAGGATACTTGGTA	2981
grl-2	T16G1.8	BC12853	TGAACTTACCACACCTGCACA	AGTCGACCTGCAGGCATGCAAGCTAAGTGCAGTTAGGATACTTGGTA	2981
grl-3	K03B8.7	BC12826	ATCCTTTCATTCGTTTCCTTCTC	AGTCGACCTGCAGGCATGCAAGCTCATCTTTCAATTTGATTCCCAAA	1798
grl-3	K03B8.7	BC12827	ATCCTTTCATTCGTTTCCTTCTC	AGTCGACCTGCAGGCATGCAAGCTCATCTTTCAATTTGATTCCCAAA	1798
grl-4	F42C5.7	BC12855	TCAGTTCGGTCTGCAAAGG	AGTCGACCTGCAGGCATGCAAGCTCCGCCTCCATTACCTCATT	1552
grl-4	F42C5.7	BC12854	TCAGTTCGGTCTGCAAAGG	AGTCGACCTGCAGGCATGCAAGCTCCGCCTCCATTACCTCATT	1552
grl-4	F42C5.7	BC15247	TTCTCGGGAAGGCTGAAGTA	AGTCGACCTGCAGGCATGCAAGCTCGAATTGAGGGATTTTGGATT	2009
grl-4	F42C5.7	TB1659	CACATTTGGAATAAGGTTAC	TATAGGATCCATCTGGAAAATAAAAACTTTTG	2214
grl-5	Y47D7A.5	BC20022	CATTGGACAAAATTTGCAGAG	AGTCGACCTGCAGGCATGCAAGCTTTCTGATCATGGCTAGCAACA	2936
grl-6	K10C2.5	BC12857	TGGTGTTGTGCCAGATTTGT	AGTCGACCTGCAGGCATGCAAGCTCAAATATGGAATGGAAGTGGAAG	940
grl-6	K10C2.5	BC12856	TGGTGTTGTGCCAGATTTGT	AGTCGACCTGCAGGCATGCAAGCTCAAATATGGAATGGAAGTGGAAG	940
grl-7	T02E9.2	BC15289	CGTCTGTTCTTTGTACGGGAG	AGTCGACCTGCAGGCATGCAAGCTCGGAATAGGATTCTGGAAATAGAA	2375

grl-8	ZC487.5	BC15274	GGGGTGGAGTCATTA GTGGA	AGTCGACCTGCAGGCATGCAAGCTCA GATCGTTAGAATACACTGCAAA	2893
grl-9	ZC487.4	BC15288	AATCGGATTCTGAAG AAGAACAAC	AGTCGACCTGCAGGCATGCAAGCTTA TCTCGATAACTATGCCAACTGC	1122
grl-10	C26F1.5	BC13003	TGTTGTTAACGGGTT TTTAAGTGA	AGTCGACCTGCAGGCATGCAAGCTTT TTAGGTGAAGTGGGTCGTG	1411
grl-10	C26F1.5	BC12880	TGTTGTTAACGGGTT TTTAAGTGA	AGTCGACCTGCAGGCATGCAAGCTTT TTAGGTGAAGTGGGTCGTG	1411
grl-11	ZK512.9	BC12881	CCCATGCATTGACAT AGCAC	AGTCGACCTGCAGGCATGCAAGCTTT TTTCAAGTTCCGCTTTTT	2847
grl-12	F28A12.2	BC20023	ACCAGGACTGTTTTG ATGGC	AGTCGACCTGCAGGCATGCAAGCTAA AGGATCGCAATGGAAAGTT	2989
grl-13	F32D1.4	BC15244	TTTTTGCCTTTTTCT TGCAT	AGTCGACCTGCAGGCATGCAAGCTCA ATGCTAAAGCTAAATGCCG	2843
grl-13	F32D1.4	BC12983	TCACGAAATTTTACG ATTTTTGC	AGTCGACCTGCAGGCATGCAAGCTTT TTTGGATTTTTGCGTTTTCT	2905
grl-14	T03D8.4	BC12985	TGGAGTACTAAGAGG CACGGTT	AGTCGACCTGCAGGCATGCAAGCTTG ATCTGTAGATTCAAAATCAATG	2899
grl-14	T03D8.4	BC15224	CAGCTGCAGTACATC CCAAA	AGTCGACCTGCAGGCATGCAAGCTTG AAAATAGGCTAAACAAGCC	2925
grl-15	Y75B8A.2 0	BC12984	TCGGAATTGAAAATA TTCGGTAA	AGTCGACCTGCAGGCATGCAAGCTGC TTTGGATTTTGTCAGACTTTTT	2888
grl-16	Y65B4BR. 6	BC12882	TTATGAAACATTGCT TTGTCGG	AGTCGACCTGCAGGCATGCAAGCTTC GTTTTTGAGAAGGCAGAAA	2840
grl-17	C56A3.1	BC15275	TTCGACGCTAAGGAC AGAAAA	AGTCGACCTGCAGGCATGCAAGCTGA GGATGCCTTGTTGGTTACA	2869
grl-17	C56A3.1	BC15276	TTCGACGCTAAGGAC AGAAAA	AGTCGACCTGCAGGCATGCAAGCTGA GGATGCCTTGTTGGTTACA	2869
grl-18	T05C3.4	BC12982	CAACTTTGGCAGCTT ATATCTCAA	AGTCGACCTGCAGGCATGCAAGCTGC AGTTGATGGAACCTGAATTT	2905
grl-18	T05C3.4	BC15230	TGTGTTGTAGATTTG AGCTCCC	AGTCGACCTGCAGGCATGCAAGCTTG CGATTGAATAGAATGTATCAG	2968
grl-19	R02D3.6	BC12981	AATGCCTGACAATAA ACGCTG	AGTCGACCTGCAGGCATGCAAGCTAC TTTTGATGTTCTATTGCTGGAT	1052
grl-19	R02D3.6	BC15228	CAAAAGTGGCAAAAA TGCAC	AGTCGACCTGCAGGCATGCAAGCTTG ATGTTCTATTGCTGGATTCAA	908
grl-20	C23H5.9	BC20095	TTCGGATTTCTAGGC CACC	AGTCGACCTGCAGGCATGCAAGCTCA GTTCCTCACCATATCAGCTTC	2754
grl-21	ZC168.5	BC15277	GCTTTTCTTTTCGGC TCCTT	AGTCGACCTGCAGGCATGCAAGCTCG ATTTCGAGTGGAATGTATGA	675
grl-23	E02A10.2	BC15278	CGGACTTGTCGCCTT TGTAT	AGTCGACCTGCAGGCATGCAAGCTTT CTGATTCTGAAAATTACGTTAGA	3059
grl-24	F11E6.2	BC12883	CAAAGCTAGGTGCTG ATGAGTTT	AGTCGACCTGCAGGCATGCAAGCTAC TTTAAATGACCTCTGTTCCTGG	2942
grl-24	F11E6.2	BC12884	CAAAGCTAGGTGCTG ATGAGTTT	AGTCGACCTGCAGGCATGCAAGCTAC TTTAAATGACCTCTGTTCCTGG	2942
grl-25	ZK643.8	BC12886	CACAATCCTCAGCTA CACTGAA	AGTCGACCTGCAGGCATGCAAGCTAA AAACCAACATTTGGGGAAC	3059
grl-25	ZK643.8	BC12885	CACAATCCTCAGCTA CACTGAA	AGTCGACCTGCAGGCATGCAAGCTAA AAACCAACATTTGGGGAAC	3059
grl-25	ZK643.8	BC15248	AGGGCGCAAAGTTCT AGGAT	AGTCGACCTGCAGGCATGCAAGCTAA CCAACATTTGGGGAACAA	1213
grl-26	K02D7.6	BC20127	TCTGAAATTCGTGCT TAGAAACTG	AGTCGACCTGCAGGCATGCAAGCTTT TGAGAATCTGAACATAAAATCC	2775

grl-27	F40C5.3	BC15279	AGAAAATTGGCCGTA ACAAC	AGTCGACCTGCAGGCATGCAAGCTGA AGGGAGGATTGGATGATGT	2956
grl-27	F40C5.3	BC15280	AGAAAATTGGCCGTA ACAAC	AGTCGACCTGCAGGCATGCAAGCTGA AGGGAGGATTGGATGATGT	2956
grl-28	T24A6.15	BC15281	CGAATTTTTCCAGAA GCCTAAAT	AGTCGACCTGCAGGCATGCAAGCTTG AGGAATGGATTATTGAATGAGA	2553
grl-29	T24A6.18	BC15282	TCTTGGTTGCAGAGT AGCCATA	AGTCGACCTGCAGGCATGCAAGCTGG GAGGAAATGATTAGCTTATGTG	1893
grl-29	T24A6.18	BC15283	TCTTGGTTGCAGAGT AGCCATA	AGTCGACCTGCAGGCATGCAAGCTGG GAGGAAATGATTAGCTTATGTG	1893
grl-31	T24A6.19	BC20024	ATAACACAGGCAAAG CGGAA	AGTCGACCTGCAGGCATGCAAGCTTT GAGGAATGGATTGTTTGATG	2364
hog-1	W06B11.4	BC15236	TACAATGTCATCGTG GCTCG	AGTCGACCTGCAGGCATGCAAGCTGT GGTGATCCAAGAGTCGGTA	3893
hog-1	W06B11.4	BC16352	GGTGCTGGTTTATTC GGAAA	AGTCGACCTGCAGGCATGCAAGCTGC CACTTGGTGTGGTCATC	2941
mom-5	T23D8.1	TB1629	GCATGCATGCGTCGT AAATCCGCAAGCAC	GCGGGATCCGATGAGAGTCGTTGATC AGC	3432
mom-5	T23D8.1	TB16230	GCATGCATGCGTCGT AAATCCGCAAGCAC	GCGGGATCCGATGAGAGTCGTTGATC AGC	3432
mom-5	T23D8.1	TB1631	GCATGCATGCGTCGT AAATCCGCAAGCAC	GCGGGATCCGATGAGAGTCGTTGATC AGC	3432
cfz-2	F27E11.3 A	TB1627	CGCTGCATGCCCTTG GAACGGGTGTCGTTG	GCGGATCCCTCACCTCCACCAGGGGA TA	10612
cfz-2	F27E11.3 A	TB1628	CGCTGCATGCCCTTG GAACGGGTGTCGTTG	GCGGATCCCTCACCTCCACCAGGGGA TA	10612
gpn-1	F59D12.4	BC20000	TCCGCGAAATAGAGG CTG	AGTCGACCTGCAGGCATGCAAGCTAA AAGCATACGAGCTTGGGTT	2972
