# Supplementary material for: Comprehensive analysis of gene expression patterns of hedgehog-related genes
Source: BMC Genomics. 2006 Oct 31;7:280. doi: 10.1186/1471-2164-7-280 (PMC1636047; doi:10.1186/1471-2164-7-280)
Supplement: Additional file 3 — Multiple sequence alignment of the Ground and Ground-like domains from C. elegans and C. briggsae. Sequences were aligned using MAFFT. Flanking sequences were trimmed to retain the Ground and Ground-like domains in the center. For GRD-1, GRD-2, and GRD-11, which each have four Ground domains, the Ground domains were extracted and added to the alignment. The alignment was displayed in Clustal_X, with the default color scheme, except for the cysteine residues, which were highlighted in yellow to visualize their conserved nature. Some Brugia malayi (Bm) sequences from previous work [4] were also included in the alignment. [file 1471-2164-7-280-S3.pdf]

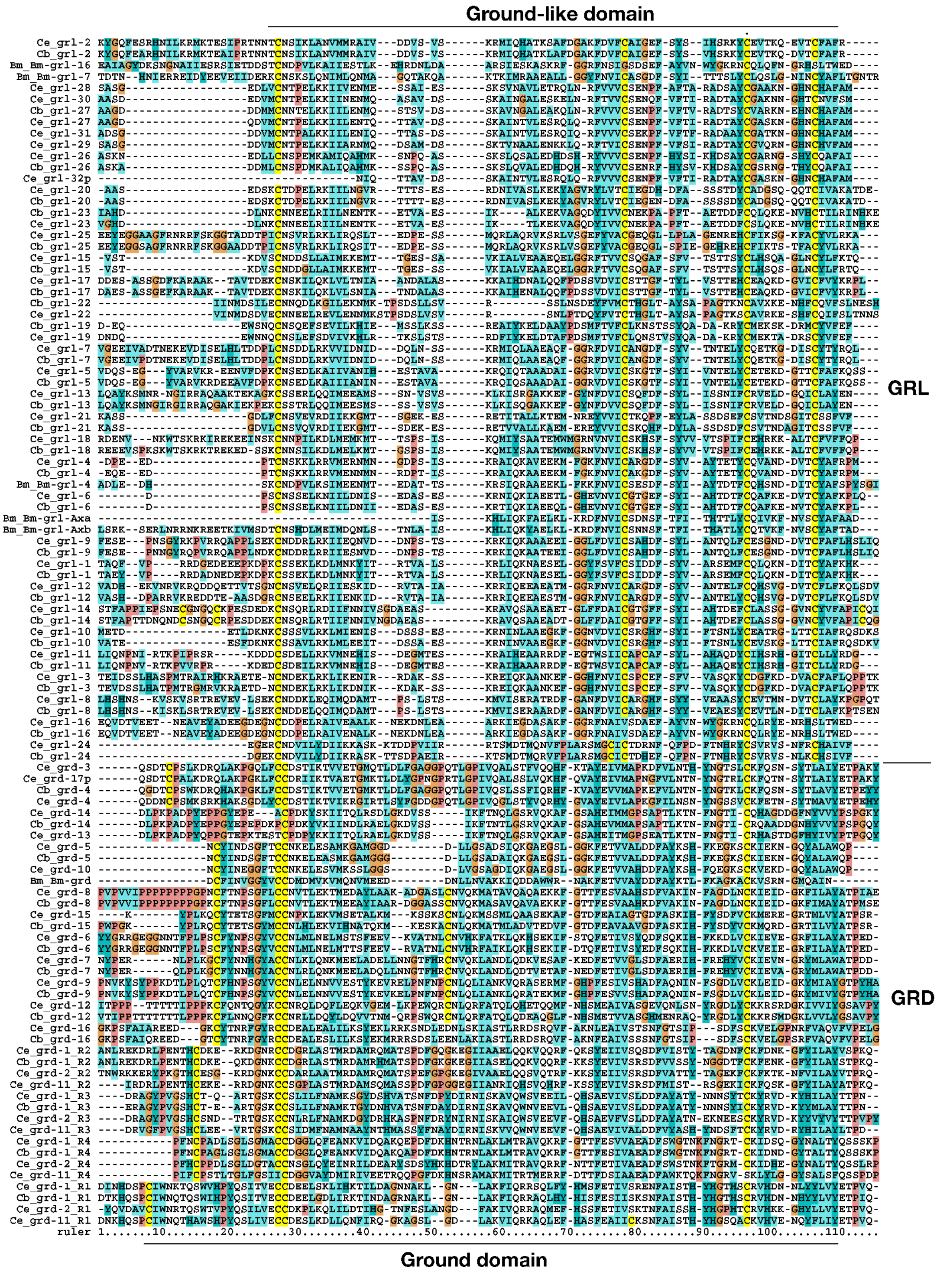

Ground-like domain
GRL
GRD
Ground domain
